# Novel Penicillin Analogues as Potential Antimicrobial Agents; Design, Synthesis and Docking Studies

**DOI:** 10.1371/journal.pone.0135293

**Published:** 2015-08-12

**Authors:** Zaman Ashraf, Abdul Bais, Md. Maniruzzaman Manir, Umar Niazi

**Affiliations:** 1 Department of Chemistry, Allama Iqbal Open University, H-8, Islamabad, Pakistan; 2 Department of Biology, College of Natural Sciences, Kongju National University, Kongju, South Korea; 3 Department of Chemistry, Kongju National University, Gongju, Republic of Korea; 4 Atta ur Rehman School of Modeling and Simulation, National University of Science and Technology, Islamabad, Pakistan; University of Edinburgh, UNITED KINGDOM

## Abstract

A number of penicillin derivatives **(4a-h)** were synthesized by the condensation of 6-amino penicillinic acid (6-APA) with non-steroidal anti-inflammatory drugs as antimicrobial agents. *In silico* docking study of these analogues was performed against Penicillin Binding Protein (PDBID 1CEF) using AutoDock Tools 1.5.6 in order to investigate the antimicrobial data on structural basis. Penicillin binding proteins function as either transpeptidases or carboxypeptidases and in few cases demonstrate transglycosylase activity in bacteria. The excellent antibacterial potential was depicted by compounds **4c** and **4e** against *Escherichia coli*, *Staphylococcus epidermidus* and *Staphylococcus aureus* compared to the standard amoxicillin. The most potent penicillin derivative **4e** exhibited same activity as standard amoxicillin against *S*. *aureus*. In the enzyme inhibitory assay the compound **4e** inhibited *E*. *coli* MurC with an IC_50_ value of 12.5 μM. The docking scores of these compounds **4c** and **4e** also verified their greater antibacterial potential. The results verified the importance of side chain functionalities along with the presence of central penam nucleus. The binding affinities calculated from docking results expressed in the form of binding energies ranges from -7.8 to -9.2kcal/mol. The carboxylic group of penam nucleus in all these compounds is responsible for strong binding with receptor protein with the bond length ranges from 3.4 to 4.4 Ǻ. The results of present work ratify that derivatives **4c** and **4e** may serve as a structural template for the design and development of potent antimicrobial agents.

## Introduction

The discovery of penicillin a β-Lactam antibiotic by Alexander Fleming in 1928 and its use into the health care system in the later phases of Second World War denotes one of the most dynamic contributions to medical science in recent history [1]. β-Lactam antibiotics have been effectively used in the treatment of infectious ailments for several years [2] and persist the most commonly utilized antibiotics due to their relatively high efficacy, low cost, ease of delivery and minimal side effects. Despite the large number of β-lactams that have already been synthesized and tested, there is still a need for new compounds of this kind [3], due to the increasing resistance of bacterial strains to certain types of anti-infectives [4].

The emergence of resistance to the major classes of antibacterial agents is recognized as a serious health concern. Particularly, the emergence of multi drug resistance strains of pathogenic bacteria is a problem of ever increasing significance reported by Kumar et al. 2010 [5]. The increasing selection for bacteria having acquired resistance mechanism progressively devaluate our antibiotic arsenal. This provides a strong incentive for continually developing novel drugs that escape the destruction of resistant bacterial strains [6]. Two mechanisms have been reported to be responsible for antibiotic resistance: structural modification in Penicillin binding protein (PBP) targets and production of β-Lactamase first identified in 1972 [7,8].

The structural modification of PBPs is a common mechanism of resistance of Gram-positive bacteria. Penicillin binding proteins (PBPs) are membrane-associated proteins that catalyze the final step of murein biosynthesis in bacteria [9]. These proteins function as either transpeptidases or carboxypeptidases and in a few cases demonstrate transglycosylase activity [10]. Both transpeptidase and carboxypeptidase activities of PBPs occur at the D-Ala-D-Ala terminus of a murein precursor containing a disaccharide pentapeptide comprising *N*-acetylglucosamine and *N*-acetyl-muramic acid-L-Ala-D-Glu-L-Lys-D-Ala-D-Ala. The Penicillins antibiotics inhibit these enzymes by competing with the pentapeptide precursor for binding to the active site of the enzyme [11]. Penicillins bind irreversibly to the active site of theses enzyme and thus prevents the final cross-linking of the peptidoglycan layer which disrupts the cell wall synthesis.

6-aminopenicillanic acid (6-APA) is an important industrial intermediate produced on large scale by the enzymatic cleavage of penicillin G (V) side-chain with penicillin amidase in solution or with immobilized enzyme. Most of the penicillins are produced by coupling 6-APA with the required side chain [12] except of Penicillin G and Penicillin V, which can be industrially produced by fermentation from high producing strains of *Penicillium chrysogenum*.

Molecular Docking is the method that predicts the preferred orientation of a drug molecule into the macromolecule and the goal is to compute the bound conformation and the binding affinity [13]. Koska et al. 2008 and Yusuf et al. 2008 also reported that docking is one of the commonly used computational methods in structure-based drug design [14,15]. The information generated from docking calculations helps to get insight into the interactions of ligands with amino acid residues in the binding pockets of targets and to predict the corresponding binding energies of ligands [16], when the experimental holo structure are unavailable [17]. Lipinski’s rule of 5 also referred rule of thumb helps in distinguishing drug likeness properties of a molecule and describes its physiochemical properties important for a drug’s pharmacokinetics in the human body [18]. These filters assist in early preclinical developments and could support to avoid costly late-stage preclinical and clinical failures [19].

In continuation of our effort in the development of effective antimicrobials agents [20,21], we report here the synthesis and docking study of novel Penicillin analogues having NSAIDs moiety using 6-APA as starting material. All of the clinically used β-lactam antibiotics (Penicillins) possess the central penam nucleus which is essential for antibacterial activity. The spectrum of activity against either Gram negative or Gram positive bacteria can be enhanced by changing the side chain amide functionality. The selected NSAIDs have different hydrophobic/hydrophilic groups and all possess −COOH group which can easily be condensed with −NH_2_ group of 6-APA to synthesize the title penicillin’s derivatives. All of the synthesized compounds **(4a-h)** were docked against penicillin binding protein (PDBID 1CEF) because of their diverse role in transpeptidases and transglycocylases. They play vital role in bacterial cell wall synthesis and inhibition of such targets may help in the development of new antibiotics. The *in vitro* antibacterial activity of synthesized penicillin derivatives was carried out against five pathogenic bacteria, two of which are Gram negative and other three are Gram positive. In this way we are able to find out the potential of our synthesized compounds against either Gram positive or Gram negative bacteria. In addition to *in vitro* antibacterial activity the enzyme inhibitory activity of compound **(4e)** was also performed against *E*. *coli* MurC, which is an important enzyme for peptidoglycan biosynthesis in bacterial cell wall.

## Materials and Methods

Melting points were recorded using a digital Gallenkamp (SANYO) model MPD 350 apparatus and are uncorrected. FTIR spectra were recorded using an FTS 3000 MX spectrophotometer; the ^1^H NMR and ^13^C NMR spectra (DMSO-*d*_6_) were recorded using a Bruker 300 MHz spectrometer. Chemical shifts (δ) are reported in ppm downfield from the internal standard tetramethylsilane (TMS). Mass spectra were performed on an Agilent 6460 Series Triple Quadrupole instrument (Agilent). The ionization was achieved by electrospray ionization in the positive ion mode (ESI+) and negative ion mode (ESI-). The capillary voltage was set to 4.0 kV. The source temperature was 120°C, and the desolvation temperature was 350°C. Nitrogen was used as a desolvation gas (flow 600 L/h). The software used for *in-silico* molecular docking studies are AutoDock Tools 1.5.6: La Jolla, CA, U.S.A., AutoDock Vina 1.1.2: La Jolla, CA, U.S.A. and Discovery Studio 4.0: San Diego, CA, U.S.A. The procedure for the synthesis of the desired compounds is depicted in Scheme I. ATP, L-alanine, AMP-PCP and bovine serum albumin (BSA) were purchased from Sigma. Malachite green phosphate detection reagent, UNAM, and E. coli MurC were prepared as described previously [22].

### General Procedure for the Synthesis of Penicillin Derivatives (4a-h)

A solution of NSAIDs having carboxylic acid group **(1a-h)** (1mmol) in dry benzene (5–8mL) was refluxed with freshly distilled thionyl chloride (1.2mmol) for 2–3 h. After the completion of reaction, excess of thionyl chloride was removed under reduce pressure to afford the acid chlorides **(2a-h)** which were dissolved in anhydrous acetone for further use. The acid chlorides **(2a-h)** were then treated with a solution of (+)-6-aminopenicillanic acid (6-APA, 1mmol) in 2% NaHCO_3_ (40mL) diluted with acetone (30 mL). The reaction mixture was stirred for 2–4h at room temperature and then concentrated under reduced pressure and washed with ethyl acetate (25mL). The aqueous layer was then acidified with HCl (0.1M), extracted with ethyl acetate and then washed with distilled water dried over anhydrous Na_2_SO_4_. The ethyl acetate was rotary evaporated and triturated with n-hexane to afford the title compounds **(4a-h)**.

#### (2S,5R,6S)-6-(3'-(4'-isobutylphenyl)propanamido)-3,3-dimethyl-7-oxo-4-thia-1-azabicyclo[3.2.0]heptane-2-carboxylic acid (4a)

Yield 78%; m.p. 115–117°C; FTIR (KBr, υ_max_ cm^-1^): 1732 (C = O β-lactam), 1667 (C = O Amide), 2954 (sp^3^C-H), 1508 (C = C); ^1^H-NMR (DMSO-*d*_6_, δ ppm): 0.93 (6H, d, *J* = 5.8 Hz, H-13’,14’), 1.20 (3H, d, *J* = 6.0 Hz, H-4’), 1.70 (3H, s, H-3”), 1.58 (3H, s, H-2”), 1.75 (1H, m, H-12), 2.36 (2H, d, *J* = 6.0, H-11), 3.43 (1H, d, *J* = 9.0, H-5), 4.3 (1H, d, *J* = 9.0, H-6), 7.15 (2H, d, *J* = 6.5 Hz, H-6’, 8’), 7.20 (2H, d, *J* = 6.5 Hz, H-5’,9’); ^13^C-NMR (DMSO-*d*_6_) δ 18 (C-13’, 14’), 22 (C-12’), 27 (C-4’), 30 (C-3’), 44 (C-11’), 70 (C-5), 72.5 (C-6), 130 (C-6’,10’), 133 (C-7’,9’), 141 (C-5’), 164 (C = O, acid), 167 (C-2’), 173 (C-7); ESI-MS: *m/z* 427 [M + 23] (M + Na)^+^.

#### (2S,5R,6S)-6-((R)-3’-(4’-isobutylphenyl)propanamido)-3,3-dimethyl-7-oxo-4-thia-1-azabicyclo[3.2.0]heptane-2-carboxylic acid (4b)

Yield 63%; m.p. 110–112°C; FTIR (KBr, υ_max_ cm^-1^): 1726 (C = O β-lactam), 1656 (C = O Amide), 2953.71 (sp^3^ C-H stretch), 1510.74(C = C) aromatic;^1^H-NMR (DMSO-*d*_6_, δ ppm): 0.91(6H, d, *J* 6.0 Hz, H-13’,14’), 1.28 (3H, d, *J* = 6.8 Hz, H-4’), 1.61 (3H, s, H-2”), 1.71 (3H, s, H-3”), 1.82 (1H, m, H-11’), 3.52(1H, q, *J* = 6.8 Hz, H-3’), 4.72(1H, s, H-2HHhhhh), 4.86 (1H, d, *J* = 9 Hz, H-5), 5.91(1H, d, J = 9 Hz, H-6), 7.0 (2H, d, J = 7.5 Hz, H-7’, 9’), 7.24 (2H, d, *J* = 7.5 Hz, H-6’, 10’); ^13^C-NMR (DMSO-*d*_6_, δ ppm): 15(C-4’), 22(C-13’,14’), 27.1(C-2”), 28(C-12’), 31.2(C-3”), 41(C-3’), 44(C-11’), 60(C-6), 64(C-2), 72(C-5), 78(C-3), 127 (C-6’, C-10’), 160(C = O, acid), 165 (C-2’), 170(C-7); ESI-MS: *m/z* 427 [M + 23] (M + Na)^+^.

#### (2S,5R,6S)-6-(3'-(7'-fluoro-[11',8']-biphenyl]-5'-yl)propanamido)-3,3-dimethyl-7-oxo-4-thia-1-azabicyclo[3.2.0]heptane-2-carboxylic acid (4c)

Yield 65%; m.p. 85–87°C; FTIR (KBr, υ_max_ cm^-1^): 1721 (C = O β-lactam), 1648 (C = O Amide), 1220 (C-F stretch); ^1^H-NMR (DMSO-*d*_6_, δ ppm): 1.20 (3H, d, *J* = 6.8 Hz, H-4’), 1.61 (3H, s, H-2”), 1.71 (3H, s, H-3”), 6.89 (1H, s, H-6’), 3.50 (1H, q, *J* = 6.8 Hz H-3’), 4.80 (1H, d, *J* = 9 Hz, H-5), 5.1 (1H, d, *J* = 9 Hz, H-6), 7.20 (1H, m, H-10’), 7.30 (1H, m, H-14’), 7.49 (2H, m, H-13’,15’), 7.50 (2H, m, H-12’ 16’), 7.72 (1H, m, H-9’); ^13^C-NMR (DMSO-*d*_6_ δ ppm): 14 (C-4’), 28 (C-2”), 30 (C-3”), 60 (C-6), 62 (C-2), 70 (C-5), 78 (C-3), 120 (C-6’), 126–128 (C-12’-16’), 130–135 (C-8’-10’), 160 (C-7’), 165 (C-5’), 168 (C = O, acid), 170 (C-2’), 171 (C-7); ESI-MS: *m/z* 465 [M + 23] (M + Na)^+^.

#### (2S,5R,6S)-6-(3'-(7'-benzoylphenyl)propanamido)-3,3-dimethyl-7-oxo-4-thia-1-azabicyclo[3.2.0]heptane-2-carboxylic acid (4d)

Yield 61%; m.p. 64°C; FT-IR (KBr,υ_max_ cm^-1^): 1733 (C = O β-lactam), 1655 (C = O Amide), 1595 (Ar-C = O), 2973 (sp^3^C-H stretch); ^1^HNMR (DMSO-*d*_6_, δ ppm): 1.20 (3H, d, *J* = 6.0 Hz, H-4’), 1.61 (3H, s, H-2”), 1.71 (3H, s, H-3”), 3.43 (1H, q, H-3’), 4.70 (1H, s, C-2), 4.74 (1H, d, *J* = 8.50 Hz, H-5), 5.0 (1H, d, *J* = 8.50 Hz, H-6), 7.50 (2H, m, H-14’, H-16’), 7.55 (1H, s, H-6’), 7.60 (1H, m, H-15’), 7.80 (2H, d, *J* = 6.5 Hz, H-13’, 17’); ^13^C-NMR (DMSO-*d*_6_, δ ppm): 15(C-4’), 28(C-3”), 30(C-2”), 40(C-3’), 60(C-6), 64(C-2), 70(C-5), 128–140(Aromatic), 165(C-acid), 170(C-2’), 173(C-7), 190(C-11’); ESI-MS: *m/z* 475 [M + 23] (M + Na)^+^.

#### (2S,5R,6S)-6-(3'-(11'-methoxynaphthalen-5'-yl)propanamido)-3,3-dimethyl-7-oxo-4-thia-1-azabicyclo[3.2.0]heptane-2-carboxylic acid (4e)

Yield 75%; m.p. 150–152°C; FT-IR (KBr, υ_max_ cm^-1^):1720 (C = O β-lactam), 1653 (C = O Amide), 1069 (C-O), 1401 (C-N); ^1^HNMR (DMSO-*d*_6_, δ ppm): 1.53(3H, s, H-4’), 1.61 (1H, s, H-2”), 1.71 (1H, s, H-3”), 3.50(1H, q, H-3’), 3.80 (3H, s, H-16’), 4.50(1H, d, *J* = 8.90 Hz, H-5), 4.70(1H, s, H-2), 5.00(1H, d, *J* = 8.90, H-6), 7.20(1H, m, H-7’), 7.22(1H, m, H-11’), 7.35–7.37(2H, m, H-6’ 10’), 7.85(2H, m, H-8’, 13’); ^13^C-NMR (DMSO-*d*_6_, δ ppm): 14(C-4’), 28(C-3”), 30(C-2”), 40(C-3’), 60(C-6), 62(C-2), 70(C-5), 78(C-3), 105(C-10’), 115(C-12’), 125–130(C-6’, 7’ 8’, 13, 14’), 133 (C-5’), 157(C-11’), 168(C-acid), 170 (C-2’), 172(C-7); ESI-MS: *m/z* 451 [M + 23] (M + Na)^+^.

#### (2S,5R,6S)-6-(3'-(4'-(20'-chlorobenzoyl)-10'-methoxy-5'-methyl-1H-indol-6'-yl) acetamido)-3,3-dimethyl-7-oxo-4-thia-1-azabicyclo[3.2.0]heptane-2-carboxylic acid (4f)

Yield 58%; m.p. 165–167°C; FT-IR (KBr, υ_max_ cm^-1^): 1723 (C = O β-lactam), 1637 (C = O Amide), 1400 (C-O stretch), 3413 (N-H stretch), 1174 (C-N stretch);^1^HNMR (DMSO-*d*_6_, δ ppm): 1.59 (3H, s, H-3”), 1.65 (3H, s, H-2”), 1.68 (3H, s, H-2”), 1.71 (3H, s, H-3”), 2.20 (3H, s, H-13’), 3.20 (2H, s, H-3’), 3.80 (3H, s, H-15’), 4.70 (1H, s, H-2), 4.80 (1H, d, *J* = 9.0 Hz, H-5), 5.00 (1H, d, *J* = 9.0 Hz, H-6), 6.30 (1H, s, H-11’), 6.60 (2H, d, *J* = 7.1 Hz, H- 19’, 21’), 7.79 (1H, m, H-9’), 7.82 (2H, d, *J* = 7.1 Hz, H-18’, 22’); ^13^C-NMR (DMSO-*d*_6_, δ ppm): 15 (C-13’), 27(C-3”),), 30 (C-3’), 32 (C-2”), 53 (C-15’), 60 (C-6), 63 (C-2), 79 (C-3), 99 (C-11’), 110 (C-9’), 130 (C-5’), 140 (C-12’), 160 (C-10’), 162 (C-7), 167 (C-16’), 168 (C = O, acid), 170 (C-5), 172 (C-2’); ESI-MS: *m/z* 581 [M + 23] (M + Na)^+^.

#### (2S,5R,6S)-6-(2-(2-((2,6-dichlorophenyl)amino)phenyl)acetamido)-3,3-dimethyl-7-oxo-4-thia-1-azabicyclo[3.2.0]heptane-2-carboxylic acid (4g)

Yield 63%; m.p 142–145°C; FT-IR (KBr,υ_max_ cm^-1^): 1729 (C = O β-lactam), 1650 (C = O Amide), 1173 (C-Cl stretch);^1^HNMR (DMSO-*d*_6_, δ ppm): 1.44 (1H, s, H-3”), 1.53 (1H, s, H-2”), 3.30 (2H, s, H-3’), 4.68 (1H, s, H-5), 4.80 (1H, s, H-6), 4.82 (1H, s, H-2), 6.50 (1H, m, H-4’), 6.70–7.00 (3H, m, H-5’ 6’, 7’), 7.07 (1H, m, H-14’),7.20 (2H, d, *J* = 6.8 Hz, H-13’, 15’),; ^13^C-NMR (DMSO-*d*_6_, δ ppm): 25 (C-3”), 28 (C-3”), 60 (C-6), 70 (C-5), 78 (C-3), 120 (C-14’), 123–125 (C-4’, 7’ 9’), 128 (C-13’, 15’), 135 (C-11’), 140 (C-12’, 16’), 163 (C-2’), 167 (C = O, acid), 172 (C-7); ESI-MS: *m/z* 517 [M + 23] (M + Na)^+^.

#### (2S,5R,6S)-6-(2-((2,3-dimethylphenyl)amino)benzamido)-3,3-dimethyl-7-oxo-4-thia-1-azabicyclo[3.2.0]heptane-2-carboxylic acid (4h)

Yield 51%; m.p 205–210°C; FT-IR (KBr,υ_max_ cm^-1^): 3472 (O-H), 1753 (C = O β-lactam), 1690 (C = O Amide), 1614 (C = C) aromatic; ^1^HNMR (DMSO-*d*_6_, δ ppm): 1.58 (3H, s, H-3”), 1.68 (3H, s, H-2”), 2.10 (3H, s, H-17’), 2.30 (3H, s, H-16’), 4.70 (1H, s, H-3), 4.86 (1H, d, *J* = 9.0 Hz, H-5), 5.15 (1H, d, *J* = 9.0 Hz, 6-CH), 6.30 (1H, m, H-15’), 6.70 (1H, m, H-13’), 6.80 (1H, m, H-14’), 7.01 (1H, m, H-7’), 8.40 (1H, m, H-5’); ^13^C-NMR (DMSO-*d*_6_, δ ppm): 20 (C-17’), 28 (C-3”), 30 (C-2”), 60 (C-6), 65 (C-3), 70 (C-5), 118–120 (7’, 5’, 13’, 3’) 125–129 (C-14’ 15’ 8’), 148 (C-4’), 168 (C-2’), 170 (C = O, acid), 172 (C-7); ESI-MS: *m/z* 462 [M + 23] (M + Na)^+^.

### Antimicrobial Study

The synthesized compounds (**4a-h)** were screened for antimicrobial activity by using agar well method against three Gram positive bacteria *Micrococcus luteus*, *Staphylococcus aureus* ATCC No. 29213, *Staphylococcus epidermidus* ATTC No. 29232 and two Gram negative bacteria *Escherichia coli* ATCC No.25922, *Salmonilla typhae* [23]. Antibacterial activity was determined by using the Mueller Hinton Agar (MHA). The fresh inoculums of these bacteria were prepared and diluted by sterilized normal saline. The turbidity of these cultures was adjusted by using 0.5Mc-Farland. A homogeneous bacterial lawn was developed by sterile cotton swabs. The inoculated plates were bored by 6 mm sized borer to make the wells. The sample dilutions were prepared by dissolving each sample (1.0mg) in 1.0 mL of DMSO used as negative control in this bioassay. The equimolar concentration of Amoxicillin (1.0mg/mL), a broad spectrum antibiotic (positive control) was prepared. These plates were incubated at 37°C for 24 hours. Antibacterial activity of penicillin derivatives **(4a-h)** was determined by measuring the diameter of zone of inhibition (mm, ± standard deviation) and presented by subtracting the activity of the negative control. The percent zone of inhibition is calculated as;

$$\begin{array}{c} \text{\%zone of inhibition }=\;\frac{\text{zone of inhibition by compound}}{\text{zone of inhibition by standard drug}}\;x\;100\\ \; \end{array}$$

### Enzymatic Assay

The enzyme inhibition assay was performed by using 6.2 nM E. coli MurC (UDP-N-acetylmuramic acid:L-alanine ligase) and 196 AM ATP, 75 AM UNAM, and 120 AM L-alanine. For IC_50_ determinations, Compound **4e** was dissolved and serially diluted in dimethyl sulfoxide (DMSO) and 2 μl added to each reaction to span a concentration range from 200 to 0.4 μM. AMP-PCP was dissolved in water and added to each reaction to span a concentration range of 2 mM to 4 μM. Reactions were incubated for 20 min at room temperature and then quenched by addition of 150 μl Malachite green phosphate detection reagent [24]. After 5 min, microtiter plates were read for absorbance at 650 nm using a Spectramax 384 Plus reader (Molecular Devices). IC_50_ values were calculated by fitting to the two-parameter equation for inhibition in GraFit 4.0 [25].

### *In-Silico* Docking

#### Ligand preparation

The two and three dimensional models of the synthesized compounds were generated using ChemBio Ultra 12 and saved as PDB format. These models may not accurately represent the atom’s location in the actual molecule and possess high energy strain at various bonds or conformational strain between atoms. To correct the models, the sketched structures were energy minimized using MM2 force field method which is an application of ChemBio 3D Ultra. This application calculates a new position of each atom so that the cumulative potential energy for the models is minimized. PDBQT files can be generated (interactively or in batch mode) and viewed using Autoduck tool (ADT) to add charges to the ligands which also automatically merged the non-polar hydrogen’s. AutoDock Vina 1.1.2: La Jolla, CA, U.S.A. uses the same PDBQT file format for molecular docking studies.

### Accession of Target Protein

Protein Data Bank (PDB) is a structural repository for biological macromolecules such as proteins and their complexes (www.rcsb.org/pdb). A serine-based penicillin binding protein (PDB entry 1CEF) with known active binding sites complexed with the drug Cefotaxime is used in this study [26]. The three dimensional structure of the target protein was retrieved from PDB by giving the PDB ID in the database. Protein Data Bank (PDB) files may have a variety of problems that need to be corrected before they can be used for docking. Before docking, the entire water molecules were removed from the protein molecule. Polar hydrogen’s were added as they are needed in the input structures to correctly type heavy atoms as hydrogen bond donor. Swiss pdb viewer (SPDV) 4.1.0 was used to minimize energy of the receptor model to eliminate unreasonable features which uses algorithm from a modeling program called GROMOS to find the nearest low energy conformation of the selected groups. The modified receptor file was then saved in the PDBQT format for docking studies.

#### Lipinski’s rule of five

Rule of five is beneficial to assess in vivo absorption abilities of the designed compounds. The newly synthesized compounds if fulfill the rule of five then it possess good oral absorption. A ligand have molar mass less than 500, hydrogen bond donors (-OH, NH) less than five, hydrogen bond acceptors (N, O) less than ten and calculated log*P* is less than five satisfy the rule of five. In the field of drug designing now a day’s rule of five has been widely applied on newly synthesized compounds for their further use as drug candidates. All of the synthesized compounds in the present study satisfy the rule of five except compound **4f** which has one violation i.e its molecular mass is greater than 500.

#### Docking Run

In order to understand the structural basis of bioactivity, structural complexes of the target enzyme with probable synthetic inhibitors were determined using computational docking approach. The AutoDock vina (http://vina.scripps.edu/) program was used to determine the binding modes of the synthetic inhibitors. AutoDock Vina uses a united-atom scoring function. It requires a specification of the “search” space in the coordinate system of the receptor, within which various position of the ligand are to be considered. The dimension of the search space was defined with Grid Center at X: -28, Y: 9, Z: 6 Å and the number of points in each dimension as X: 25, Y: 25, Z: 25 and Spacing (Angstrom): 0.3750. AutoDock Vina runs in the command prompt mode and millions of docked positions being analyzed. Each output file has several models ranked in the descending order in terms of binding energy. The predicted binding affinity of the ligand with target protein is represented in kcal/mole. In each case only the best mode is usually selected and used for subsequent analysis.

## Results and Discussion

### Chemistry

**Fig 1:**
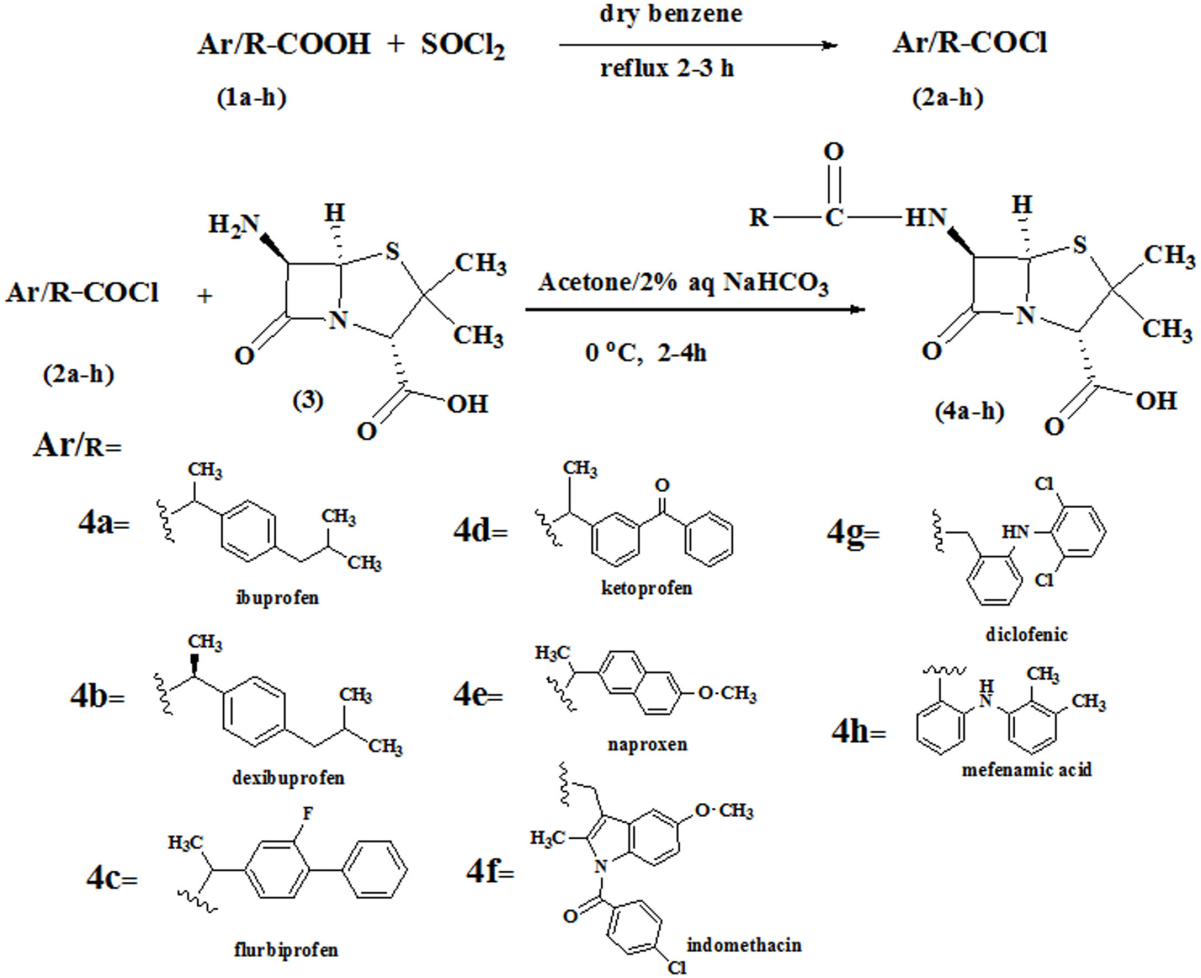
Synthesis of Penicillin derivatives (4a-h).

Penicillin derivatives **(4a-h)** have been synthesized by following the preciously described method [27] with slight modification shown in Fig 1. The acid chlorides **(2a-h)** of NSAIDs have been synthesized in the first step which in the second step was then condensed with the 6-aminopenicillinic acid to afford the final products. The title compounds **(4a-h)** were synthesized by simple nucleophilic substitution of halogen in acid chlorides by amino group of 6-aminopenicillinic acid. The structures of all of the synthesized penicillines derivatives were confirmed by FTIR, ^1^H NMR and ^13^C NMR spectroscopic data. The carbonyl of the beta lactam appeared between 1720–1753cm^-1^ is more deshielded than the carbonyl of the amide functionality which appeared at 1637–1690 cm^-1^ in FTIR spectrum. This is because of the strain of the beta lactam ring. In the ^13^C NMR spectral data of compounds **(4a-h)** the beta lactam carbonyl appeared at 170–175ppm more down field than the amide carbonyl which appeared at 160–168ppm.

### Antimicrobial Activity

**Fig 2:**
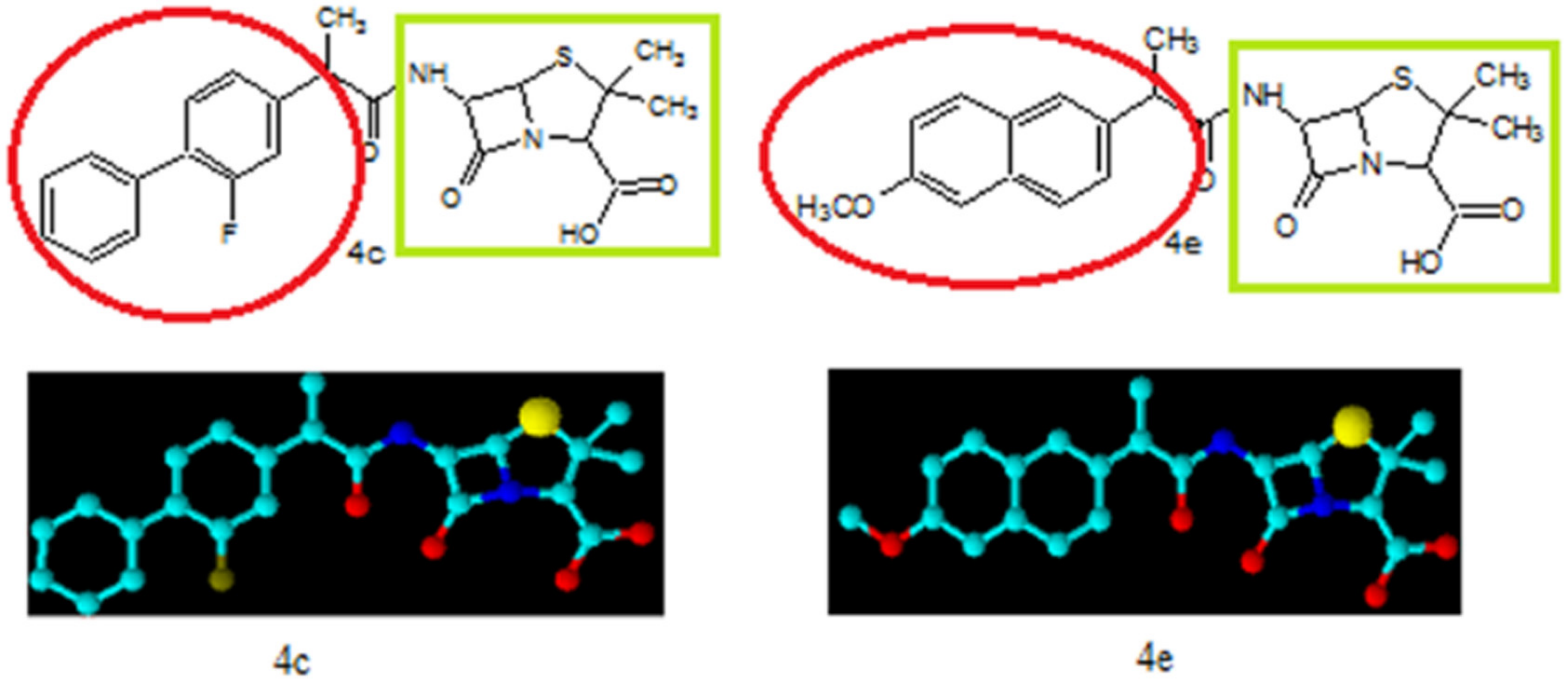
Chemical structure and three dimensional view of compounds 4c and 4e with highlighted green show the main penicillin nucleus and red show the designed moiety to increase the bioactivity.

The synthesized penicillin’s derivatives **(4a-h)** were screened for their antibacterial activity against five different bacteria, *Salmonilla typhae* and *Micrococcus luteus* are clinical isolates, rest of three are pathogenic and their ATTC numbers are provided. The bioactivity of synthesized compounds is presented in zone of inhibition of bacterial growth in millimeter (mm). The Amoxicillin a penicillin derivative was used as positive control to compare the antibacterial potential of our synthesized penicillins derivatives. The antibacterial activity results indicated that compounds **4a, 4f** and **4h** exhibited 60%, 72% and 64% bacterial zone inhibition against *E*. *coli* respectively. On the other hand **4a, 4b** and **4h** displayed 56%, 60% and 68% inhibitory activity against *S*. *aureus* respectively. The excellent zone inhibition was shown by the compound **4c** and **4e**. The central penam nucleus in all of these compounds is same but the side chain functionality is different as highlighted in Fig 2 which is responsible for difference in bioactivity among these compounds.

The compound **4e** exhibited 100% growth inhibition against *Staphylococcus aureus* while compound **4c** showed 76% of zone inhibition when compared with the standard. The compound **4e** showed 80% and 76% of zone inhibition against *Staphylococcus epidermidus* and *Escherichia coli* respectively but compound **4c** displayed 80% of zone inhibition against both *Staphylococcus epidermidus* and *Escherichia coli*. The compound **4e** possess methoxy substituted naphthyl moiety as side chain functionality which may play very significant role in antibacterial activity. In case of compound **4c** the fluorine substituted biphenyl ring system is present which is responsible for its high antibacterial potential. We found that *Micrococcus luteus* is more resistant among the tested bacteria in this study against all of the synthesized compounds and the standard drug Table 1. The percent zone of inhibition was calculated on the basis of activity of the reference drug (see experimental part for exact formula). The central penam nucleus is common in both the compounds **(4c)** and **(4e)** but the side chain amide R group play significant role in antibacterial potential. Both these compounds also differ in degree of hydrophobicity because of different functionalities. The side chain R group contains two phenyl rings in both these derivatives but in case of compound **(4e)** phenyl rings are fused while in compound **(4c)** two independent phenyl rings are present. These structural features explain the difference in the antibacterial potential among the two compounds.

**Table 1:** Antibacterial activity result of penicillin derivatives (4a-h).

Zone of inhibition(mm ± standard deviation)					
Codes	*E*.*c*.	*S*.*t*.	*S*.*e*.	*S*.*a*.	*M*.*l*.
**4a**	30±0.13	-	12±0.09	28±0.25	-
**4b**	-	-	-	30±0.19	-
**4c**	40±0.16	-	24±0.06	38±0.08	-
**4d**	30±0.20	-	14±0.17	0	-
**4e**	38±0.18	-	24±0.21	50±0.18	-
**4f**	36±0.09	-	2±0.12	4±0.05	-
**4g**	10±0.11	-	14±0.08	16±0.15	-
**4h**	32±0.16	-	2±0.22	34±0.13	-
**Amoxicillin**	50±0.06	36±0.16	30±0.08	50±0.18	8±0.05

Data given are mean of three replicates± standard error. Activity presented in millimeters (mm). (-) = No activity measured. *Escharichia coli* **(*E*.*c*.)**, *Salmonilla typhae* **(*S*.*t*.)**, *Staphylococcus epidermidus* **(*S*.*e*.)**, *Staphylococcus aureus* **(*S*.*a*.)**, *Micrococcus luteus* **(*M*.*l*.)**

#### Enzyme Inhibition Assay

The *E*. *coli* MurC (UDP-N-acetylmuramic acid:L-alanine ligase) is an essential enzyme in bacteria for peptidoglycan biosynthesis. It catalyzes the conversion of UDP-MurNAc to UDP-MurNAc-Ala in the assembly of the disaccharide-peptide unit required for peptidoglycan. Thus inhibitors of MurC are the potential candidates for the development of potent antibacterials. The title compound **4e** was selected to perform *E*. *coli* MurC inhibitory activity using the Malachite assay for phosphate detection. Compound **4e** showed an IC_50_ of 12.5 μM compared to 17 μM for AMP-PCP, a non-hydrolyzable ATP analogue used as a control inhibitor.

### *In-Silico* Docking Studies

Molecular docking is a virtual substitute of the x-ray crystallographic study of the drug binding to the target protein/DNA. In X-rays crystallography, the crystals of the enzyme is placed in the solution of the drug for binding to the active site and the resultant complex is then analyzed by crystallographic method to explore the structure activity relationship. The same result can be obtained by Molecular docking which involve the virtual complexing of the drug candidates in the active site of the crystallographic structure of target protein to predict the structure activity relationship. First of all the active binding sites in penicillins binding protein (1CEF) were identified, these sites are important in molecular docking and de novo drug design. The volume of the binding pocket was identified using Pocket Finder (http://www.modelling.leeds.ac.uk/qsitefinder/) and the binding site volume was found to be 240 Cubic Angstroms while the total volume of the protien was 31978 Cubic Angstroms. The binding pocket obtained from this study is essentially the same as that seen in the crystal structure of serine-based D-alanyl-D-alanine carboxypeptidase/transpeptidase (PDB1CEF). The active binding site located in a cleft between the five-stranded anti-parallel β sheet and the large α helical cluster S1 Fig. The S1 Table represents the various types of interactions between compounds and penicillin binding protein (1CEF) functionalities.

The conserved amino acid residues also termed as super-sites were then identified as it is functionally more important for kinetics and thermodynamics of protein folding than the non-conserved amino acid residues. Earlier studies have highlighted that Ser62 present in the active site binds directly to penicillin like drugs. This residue is conserved among all twenty one homolog’s identified indicating the importance of the Ser62. S2 Fig showed the conserved amino acid residue in the receptor protein. Residues Asn161, Ser 130, His 298, Thr 299 and Lys 65 which lies within the binding site are highly conserved and may play a major role in substrate binding or catalysis. Asn161 is conserved among eighteen of the twenty one homologs identified. The hallmark of all serine based penicillin interacting proteins is the presence of three well conserved motifs in the active site, the SXXK, (S/Y)XN and KT(S)G triads. All the Penicillin analogues docked with PBP using AutoDock vina gives lowest energy complexes stabilized by intermolecular hydrogen bonds and π-π stacking interactions. The interactions in these complexes vary depending on the size, linkage and the functional groups. These stacking interactions have been proposed as the reason for the increased binding affinities of these larger inhibitors. Hydrogen bonding contributes most to the binding affinities of all penicillin derivatives with the receptor protein. Table 2 presented the interacting parts of the ligands and the amino acids of receptors along with number and bond length of hydrogen bonds.

**Table 2:** Hydrogen bonding between penicillin derivatives (4a-h) and receptor.

Ligandcodes	Length of H-bond(Å)	Interacting ligand parts	Interacting Amino acid	Side chain/Backbone^a^ Amino acid involve in H-bonding	No. of H-Bonds
**4a**	3.4	C = O(carboxyl)	Thr 123	Side chain	3
	4	C = O (Sec-NH)	Thr 116	Side chain	
	5	C = O (Sec-NH)	Asn 161	Side chain	
**4b**	3.8	C = O(carboxyl)	Asn 327	Backbone	2
	4	C = O (Sec-NH)	Tyr 116	Side chain	
**4c**	4.2	C = O(carboxyl)	Asn 327	Backbone	3
	3.7	C = O(carboxyl)	Ser 236	Side chain	
	3.5	Hydrogen(Carboxyl)	Ser 236	Side chain	
**4d**	4	C = O(side chain)	Ser 326	Side chain	5
	4.1	C = O(Sec-NH)	Thr 116	Side chain	
	5.6	C = O(Sec-NH)	Asn 161	Side chain	
	4.2	C = O(B-lactam)	Thr 301	Side chain	
	4.3	C = O(carboxyl)	Thr 301	Side chain	
**4e**	5.2	C = O(Sec-NH)	Asn 161	Side chain	3
	3.6	C = O(Sec-NH)	Thr 116	Side chain	
	3.9	C = O(carboxyl)	Thr 123	Side chain	
	7.5	O-CH_3_	Arg 285	Side chain	
**4f**	6.5	C = O(side chain)	Arg 285	Side chain	2
	4.1	C = O(carboxyl)	Arg 303	Side chain	
**4g**	5.2	C = O(Sec-NH)	Asn 161	Side chain	5
	4.3	C = O(Sec-NH)	Thr 116	Side chain	
	3.8	C = O (B-lactam)	Thr 301	Side chain	
	3.8	OH(Carboxyl)	Asn 327	Backbone	
	4.4	C = O(carboxyl)	Ser 326	Side chain	
**4h**	4	C = O(Sec-NH)	Thr 116	Side chain	5
	5.3	C = O(Sec-NH)	Asn 161	Side chain	
	3.7	C = O (B-lactam)	Thr 301	Side chain	
	4.2	C = O(carboxyl)	Asn 327	Backbone	
	4.1	C = O(carboxyl)	Ser 326	Side chain	

^a^Amino acid main chain comprising of NH_2_-CH_2_-COOH.

**Fig 3:**
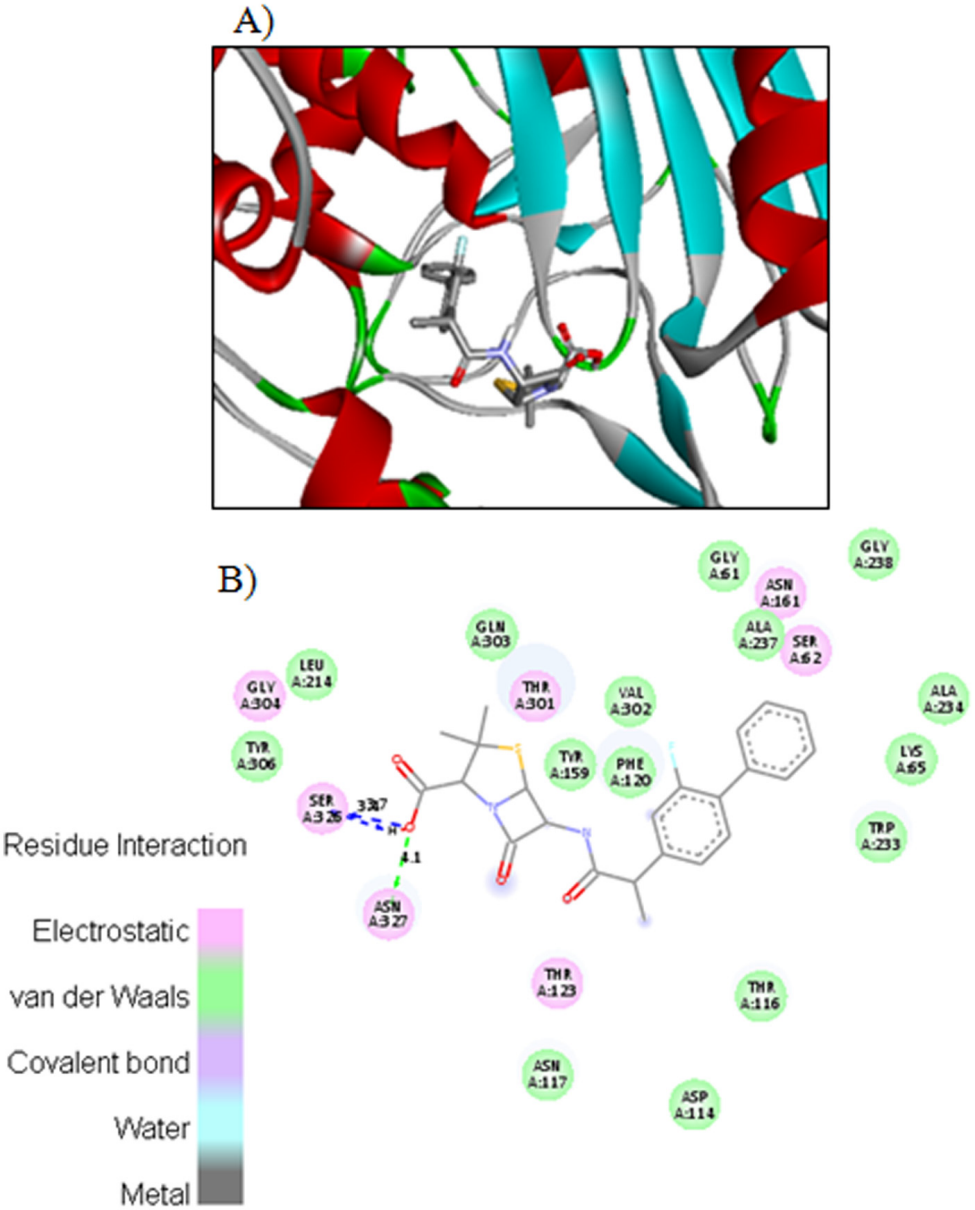
The potential ligand-protein interactions of compound 4c with the active site of Penicillin binding protein (PDB ID 1CEF) generated by using Discovery Studio 4.0. A) The three-dimensional docking of the compound **4c** in the binding pocket. B) The two dimensional interactions of **4c** with amino acid residues are shown as balls colored by the type of interaction.

**Fig 4:**
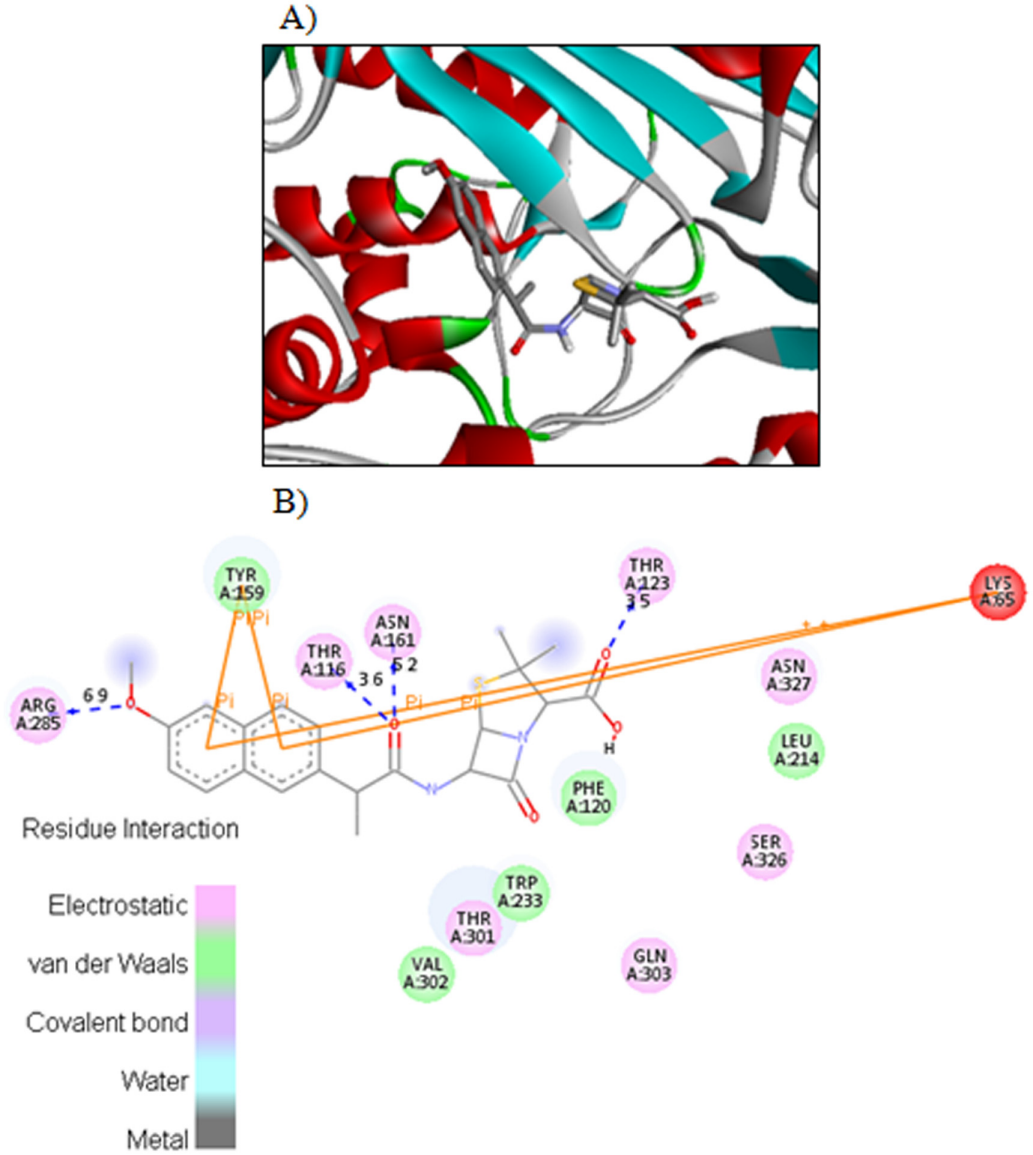
The potential ligand-protein interactions of compound 4e with the active site of Penicillin binding protein (PDB ID 1CEF) generated by using Discovery Studio 4.0. A) The three-dimensional docking of the compound **4e** in the binding pocket. B) The two dimensional interactions of **4e** with amino acid residues are shown as balls colored by the type of interaction.

The carboxyl oxygen in compound **4c** involved in hydrogen bonding with amino acids ASN327 and SER236 with bond length 4.2 Ǻ and 3.7 Ǻ respectively (Fig 3). The carboxyl hydrogen in compound **4c** also interacts with side chain SER236 through hydrogen bond having bond length 3.5 Ǻ. Fig 4 displayed the two and three dimensional ligand-protein interactions of compound **4e** with the active binding sites of penicillins binding protein. It was found from the figure 8 that compound **4e** formed π-π stacks between naphthyl ring of the inhibitor and TYR159 and LYS65 of PBP. The carboxyl oxygen involved in the hydrogen bonding with THR123 having bond length 3.9 Ǻ. The methoxy oxygen in the same compound interacts with ARG285 and secondary amide oxygen bind with side chain amino acids ASN161 and THR116 with bond length 5.2 and 3.6 Ǻ respectively. The binding affinities calculated for compounds **4c** and **4e** are 8.8 and 8.9Kcal/mol respectively. The most potent penicillin’s derivatives also showed good docking scores.

The side chain indole moiety in case of compound **4f** show π-π stacks interactions with TYR159 and LYS65. On the other hand carboxylic oxygen and amide carbonyl oxygen in compound **4f** forms hydrogen bonds with GLN303 and ARG285 having bond length respectively. Among all of the synthesized penicillin derivatives compound **4f** gives the lowest energy complex with binding energy of -9.2kcal/mol. The β-lactam carbonyl carbon of **4d, 4g**, and **4h** also forms H-bonds at the interacting distances of 4.2, 3.8 and 3.7 Ǻ respectively. Two and three dimensional potential ligand-protein interactions of synthesized penicillin derivatives **(4a, 4b, 4d, 4f, 4g and 4h)** with the active site of Penicillin binding protein (PDB ID 1CEF) are shown in S3 to S8 Figs respectively as supporting information. All of the synthesized compounds evaluated for their docking orientation to PBP exhibited reasonable affinity with good dock score. The binding energies of all these penicillin derivatives in the most favorable conformation are given in Table 3.

**Table 3:** The binding Affinities of the penicillin derivatives (4a-h).

Compounds	Mode	Binding Affinity(kcal/mol)
**4a**	1	-7.8
**4b**	1	-7.8
**4c**	1	-8.8
**4d**	1	-8.8
**4e**	1	-8.9
**4f**	1	-9.2
**4g**	1	-8.2
**4h**	1	-9.0

### Lipinski’s Rule of Five

The synthesized compounds were tested for Lipinski’s Rule of 5 using the Molinspiration server (http://www.molinspiration.com). The inputs were given as a SMILES string. The calculated *logP* values and other structural properties of synthesized penicillin analogues **(4a-h)** are shown in Table 4. The results of the calculations for the molecules designed in this study show that all molecules have a potential for good in vivo absorption, since all the compounds shows zero voilation of the rule, except for single one in case of **4f** whose molecular weight exceed the allowed range.

**Table 4:** Lipinski’s Rule of Five screening data for penicillin derivatives (4a-h).

Ligandcodes	Lipinski's Rule of Five(Molecular properties)							
	miLogP	Molar refractivity	No. of Atoms	Molar Mass	H-bond acceptor	H-bond donor	No. of rotatable bonds	No. of violations
**Rule**	**<5**	**40–130**	**20–70**	**<500**	**<10**	**<5**	**≤10**	
**4a**	3.917	86.706	28	404.53	6	2	6	0
**4b**	3.917	86.706	28	404.53	6	2	6	0
**4c**	4.503	86.706	31	442.05	6	2	5	0
**4d**	4.047	103.77	32	452.53	7	2	6	0
**4e**	3.832	95.94	30	428.51	7	2	5	0
**4f**	4.44	117.945	38	556.04	9	2	6	1
**4g**	4.953	98.733	31	480.37	7	3	5	0
**4h**	4.47	98.733	31	439.53	7	3	5	0

## Conclusions

In conclusion we describe the synthesis and antimicrobial screening of novel penicillin analogues incorporating NSAIDs moiety as potent antibacterial agents. *In-silico* docking of these derivatives with Penicillin binding protein (PDBID 1CEF) was performed in order to predict their binding affinity. The title compounds **4c** and **4e** showed remarkable activity against *E*. *coli*, *S*. *epidermidus* and *S*. *aureus* with good docking scores. The title compound **4e** also showed good enzyme inhibitory activity against *E*. *coli* MurC with IC_50_ 12.5μM. All of the synthesized derivatives **(4a-h)** exhibited high binding affinity with binding energies between -7.8 to 9.2kcal/mol. The carbonyl oxygen of carboxyl functionality in all molecules was involved in H-bonding with active site residues of target with the bond length ranges from 3.4 to 4.4 Ǻ. Similarly, the carbonyl oxygen of the secondary amide forms H-bonds in all molecules with the exception of **4f**. Among the compounds tested for docking study, **4f** showed high affinity with low energy of -9.2kcal/mol with employed protein. The clinical isolates *Micrococcus luteus* and *Salmonilla typhae* were found to be most resistant against all of synthesized compounds and standard also. Our results endorse us that compounds **4c** and **4e** may serve as a structural template for the design and development of highly potent antimicrobial agents.

## Supporting Information

S1 Fig Image showing the position of the active site occupied between five-stranded anti-parallel β-sheet and the large α-helical cluster. (TIF)

S2 Fig The conserved amino acids residues of the receptor protein. (TIF)

S3 Fig The potential ligand-protein interactions of compound 4a with the active site of Penicillin binding protein (PDB ID 1CEF) generated by using Discovery Studio 4.0. A) The three-dimensional docking of the compound **4a** in the binding pocket. B) The two dimensional interactions of **4a** with amino acid residues are shown as balls colored by the type of interaction.
(TIF)

S4 Fig The potential ligand-protein interactions of compound 4b with the active site of Penicillin binding protein (PDB ID 1CEF) generated by using Discovery Studio 4.0. A) The three-dimensional docking of the compound **4b** in the binding pocket. B) The two dimensional interactions of **4b** with amino acid residues are shown as balls colored by the type of interaction.
(TIF)

S5 Fig The potential ligand-protein interactions of compound 4d with the active site of Penicillin binding protein (PDB ID 1CEF) generated by using Discovery Studio 4.0. A) The three-dimensional docking of the compound **4d** in the binding pocket. B) The two dimensional interactions of **4d** with amino acid residues are shown as balls colored by the type of interaction.
(TIF)

S6 Fig The potential ligand-protein interactions of compound 4f with the active site of Penicillin binding protein (PDB ID 1CEF) generated by using Discovery Studio 4.0. A) The three-dimensional docking of the compound **4f** in the binding pocket. B) The two dimensional interactions of **4f** with amino acid residues are shown as balls colored by the type of interaction.
(TIF)

S7 Fig The potential ligand-protein interactions of compound 4g with the active site of Penicillin binding protein (PDB ID 1CEF) generated by using Discovery Studio 4.0. A) The three-dimensional docking of the compound **4g** in the binding pocket. B) The two dimensional interactions of **4g** with amino acid residues are shown as balls colored by the type of interaction.
(TIF)

S8 Fig The potential ligand-protein interactions of compound 4h with the active site of Penicillin binding protein (PDB ID 1CEF) generated by using Discovery Studio 4.0. A) The three-dimensional docking of the compound **4h** in the binding pocket. B) The two dimensional interactions of **4h** with amino acid residues are shown as balls colored by the type of interaction.
(TIF)

S1 Table Descriptions of the various types of interactions between inhibitors and target protein functionalities shown in figures. (DOCX)
