# Supplementary figures and images for: Novel Penicillin Analogues as Potential Antimicrobial Agents; Design, Synthesis and Docking Studies
Source: PLoS One. 2015 Aug 12;10(8):e0135293. doi: 10.1371/journal.pone.0135293 (PMC4534092; doi:10.1371/journal.pone.0135293)

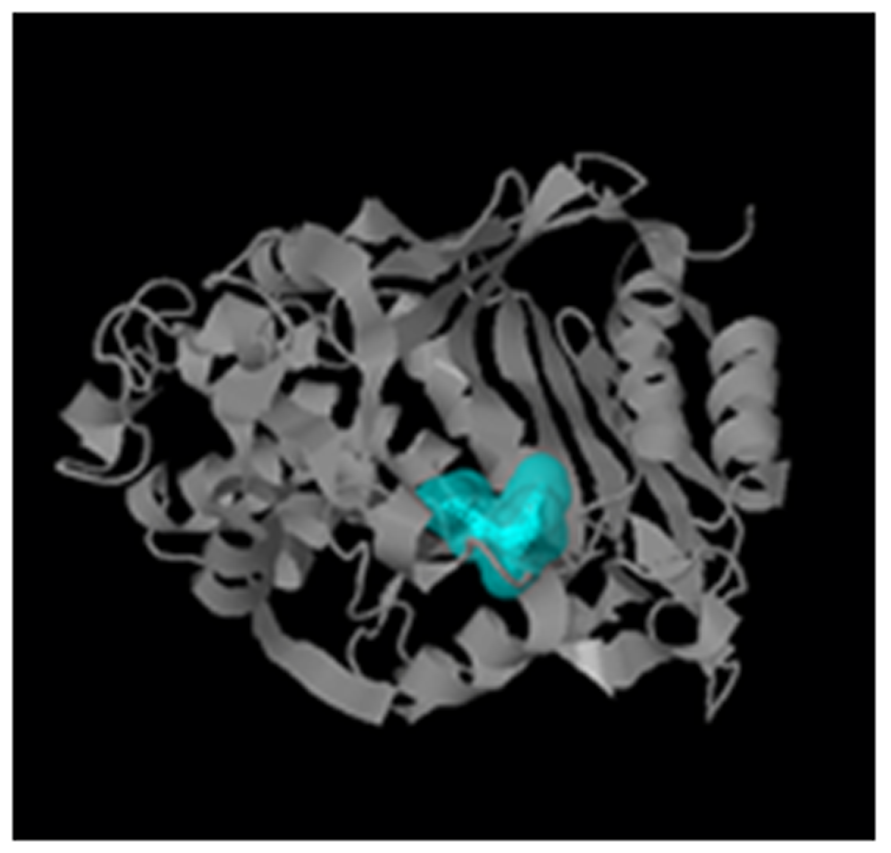

Supplement: S1 Fig — (TIF) [file pone.0135293.s001.tif]

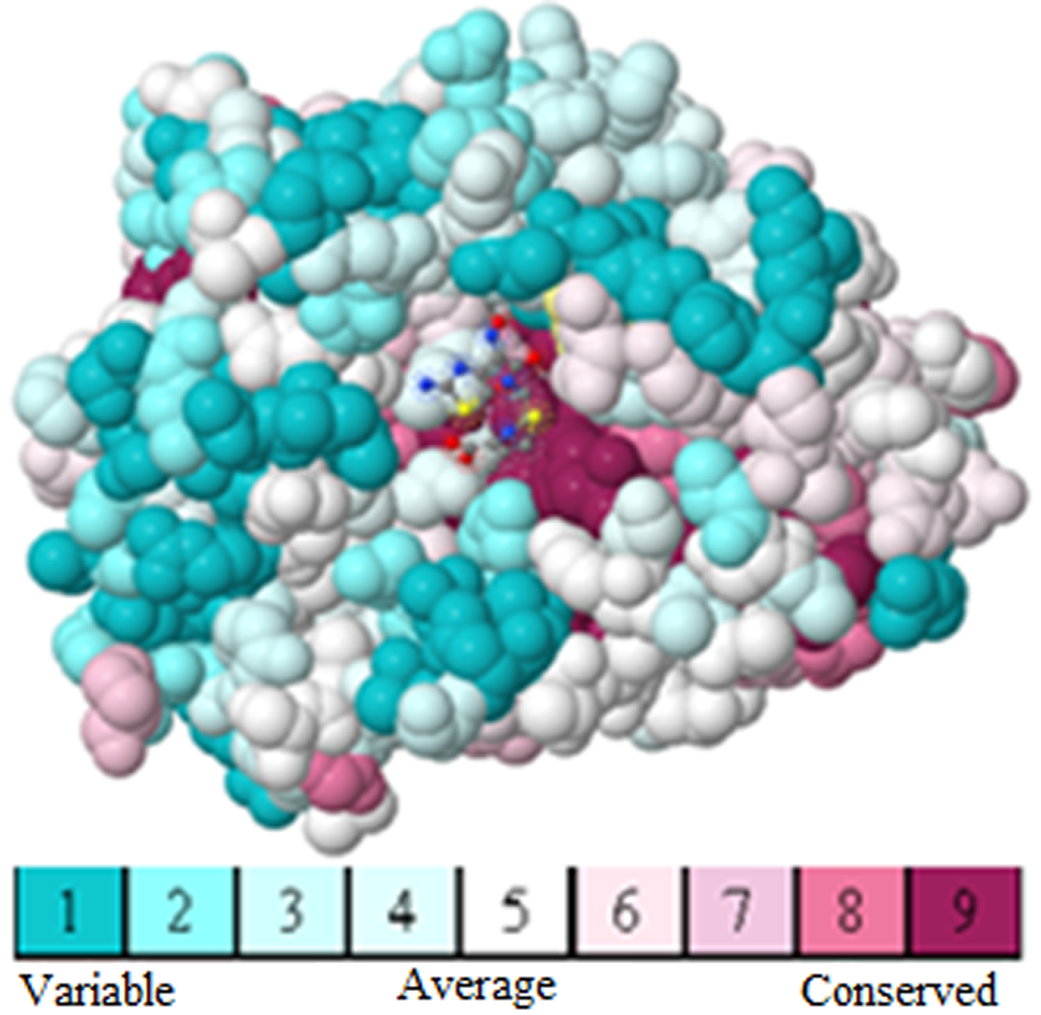

Supplement: S2 Fig — (TIF) [file pone.0135293.s002.tif]

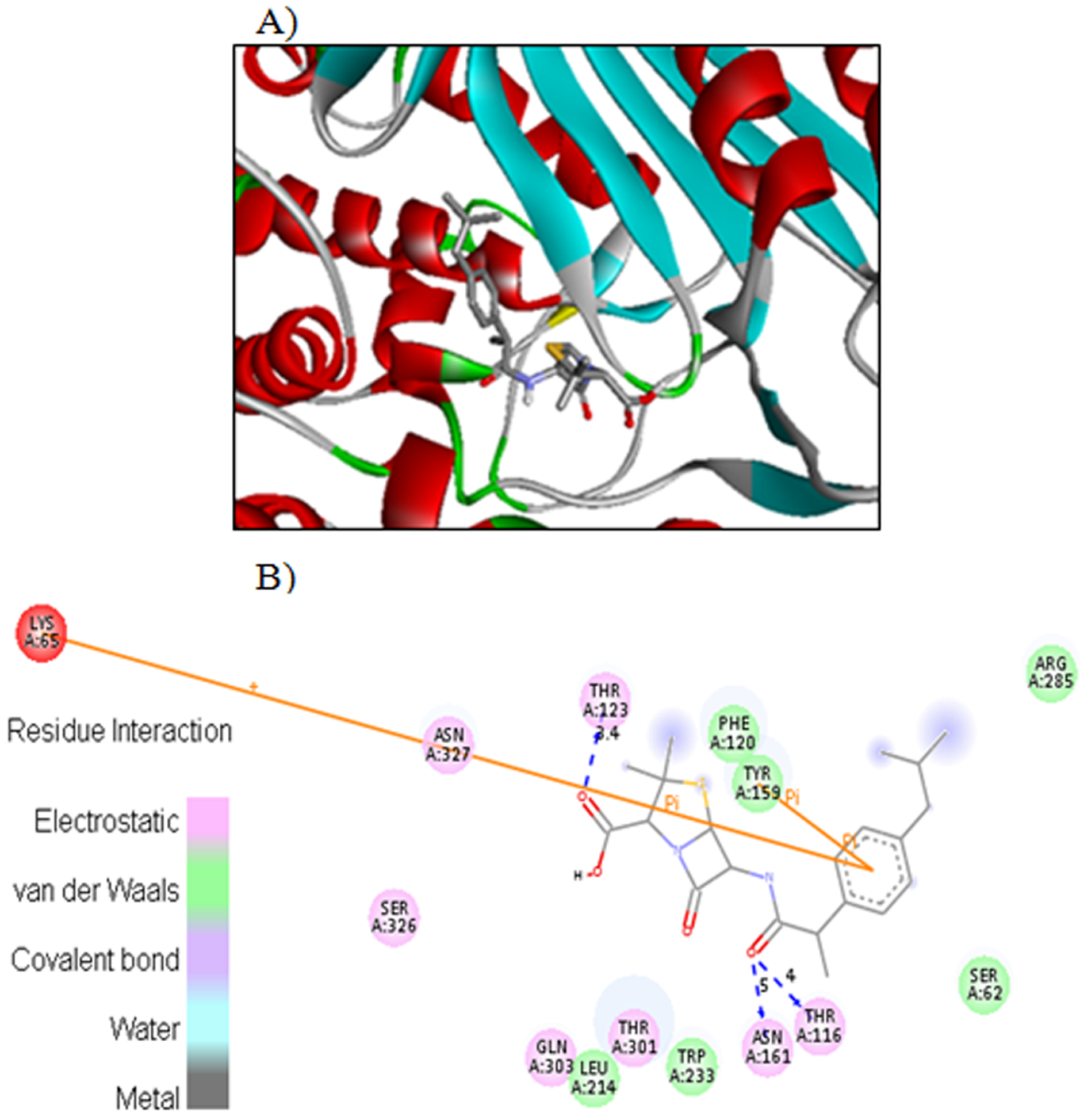

Supplement: S3 Fig — A) The three-dimensional docking of the compound 4a in the binding pocket. B) The two dimensional interactions of 4a with amino acid residues are shown as balls colored by the type of interaction. (TIF) [file pone.0135293.s003.tif]

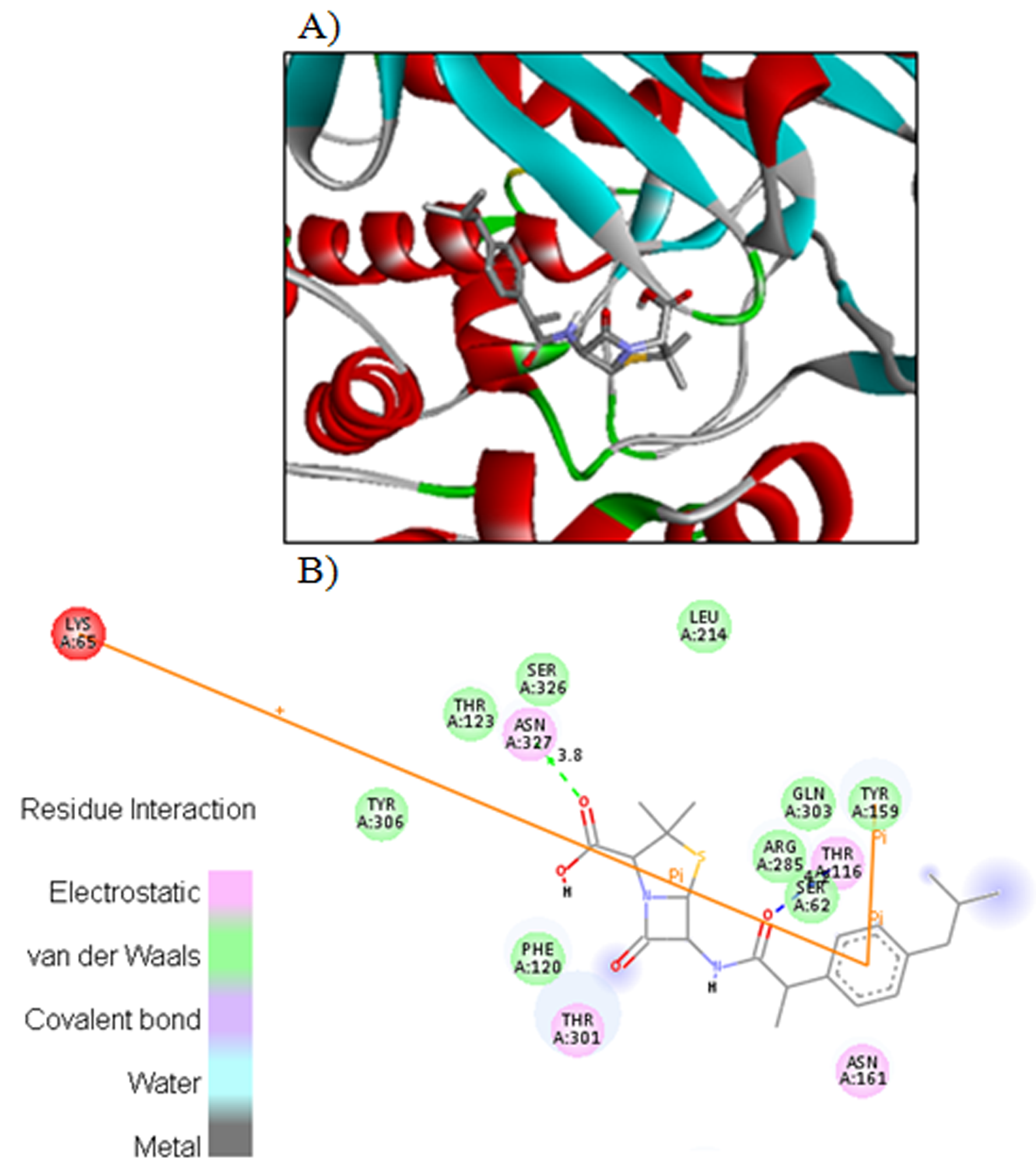

Supplement: S4 Fig — A) The three-dimensional docking of the compound 4b in the binding pocket. B) The two dimensional interactions of 4b with amino acid residues are shown as balls colored by the type of interaction. (TIF) [file pone.0135293.s004.tif]

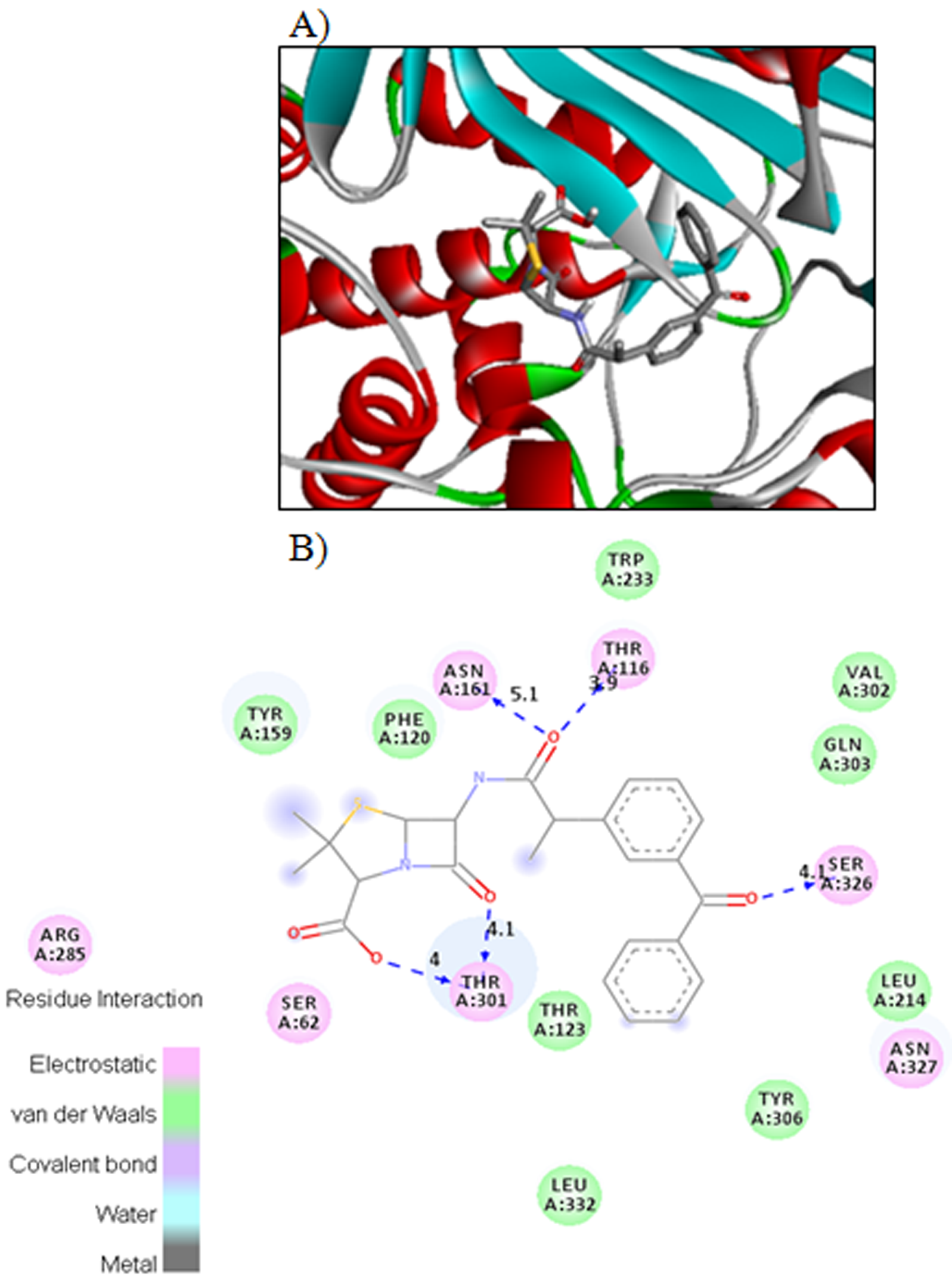

Supplement: S5 Fig — A) The three-dimensional docking of the compound 4d in the binding pocket. B) The two dimensional interactions of 4d with amino acid residues are shown as balls colored by the type of interaction. (TIF) [file pone.0135293.s005.tif]

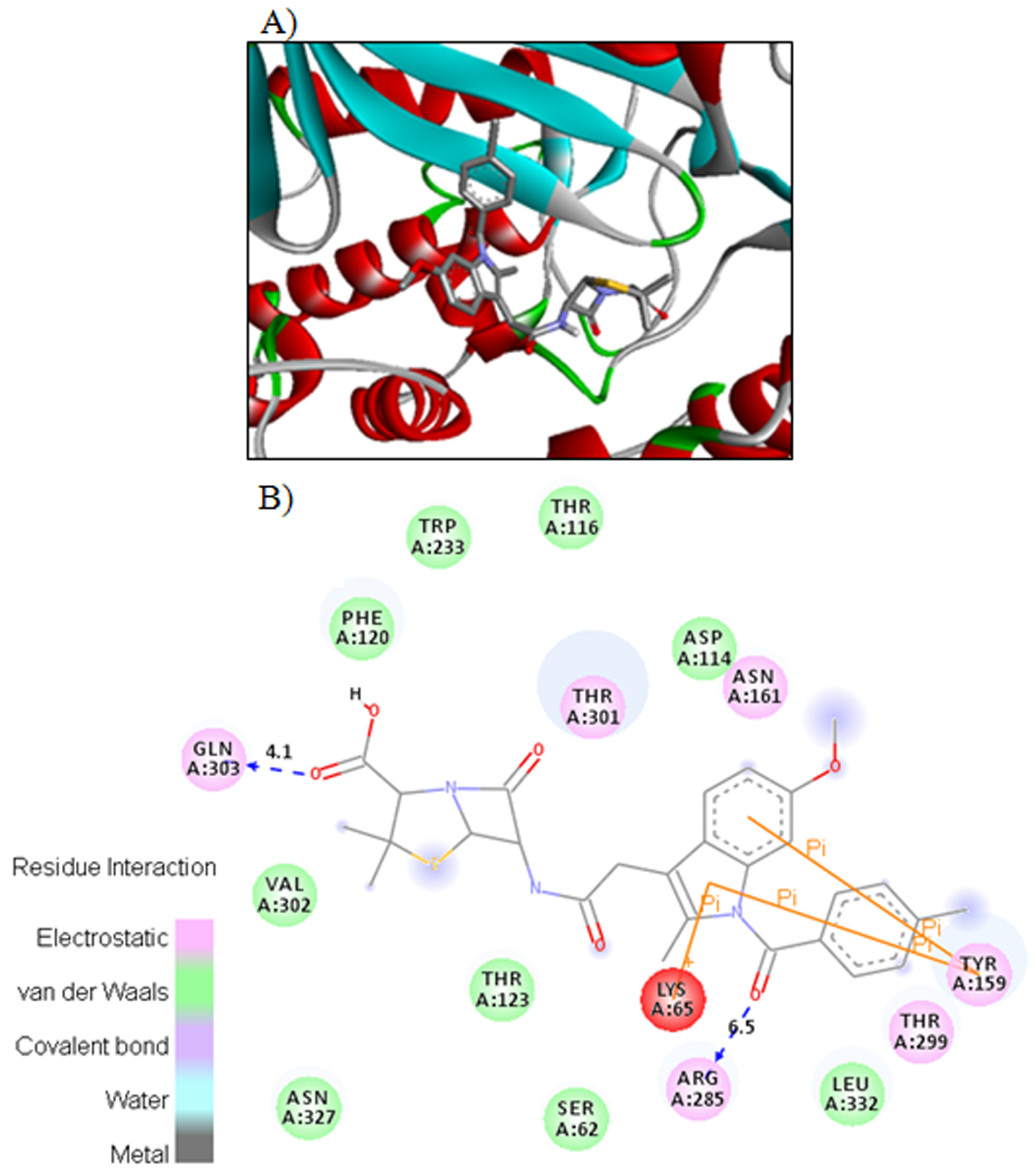

Supplement: S6 Fig — A) The three-dimensional docking of the compound 4f in the binding pocket. B) The two dimensional interactions of 4f with amino acid residues are shown as balls colored by the type of interaction. (TIF) [file pone.0135293.s006.tif]

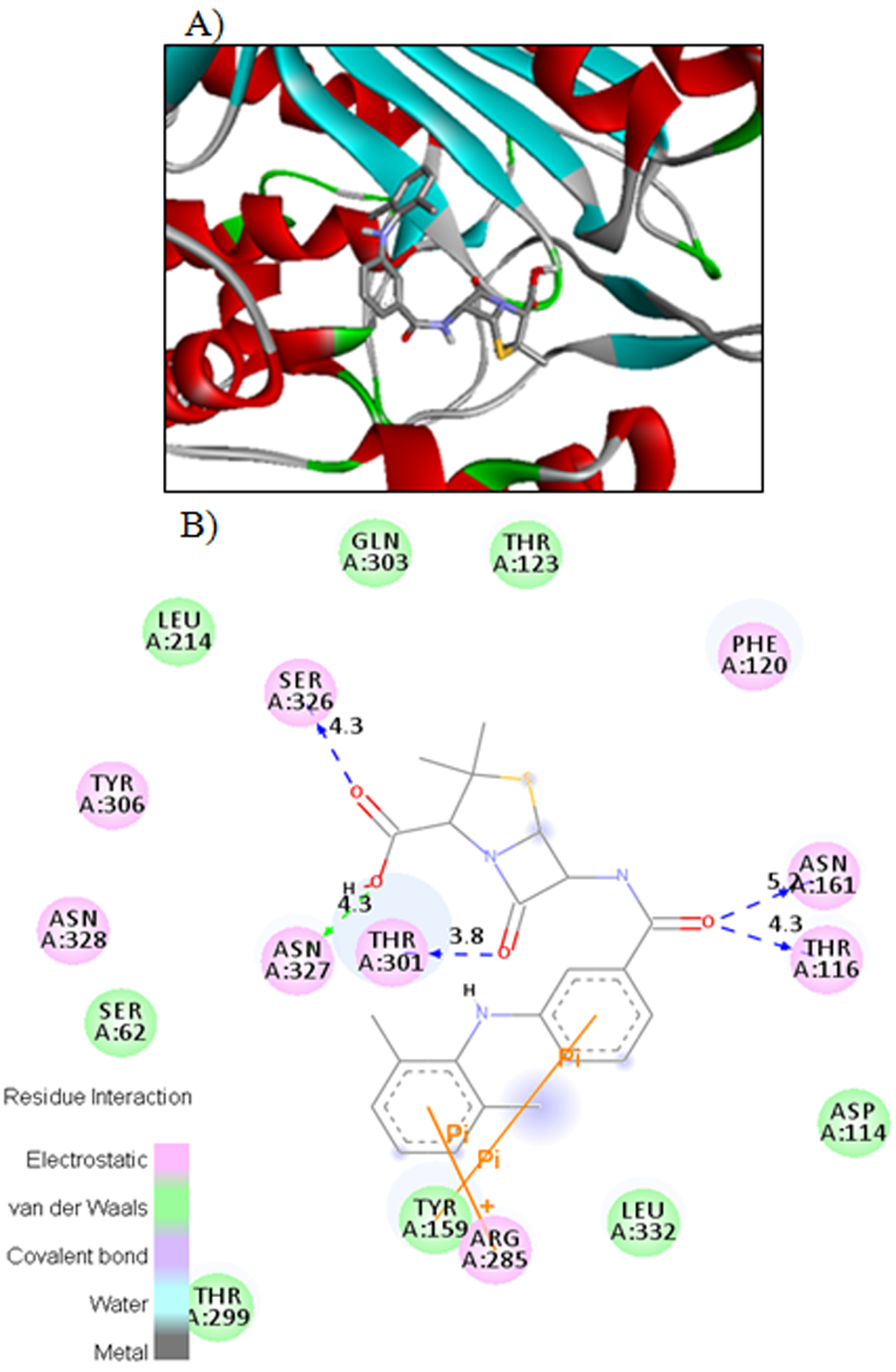

Supplement: S7 Fig — A) The three-dimensional docking of the compound 4g in the binding pocket. B) The two dimensional interactions of 4g with amino acid residues are shown as balls colored by the type of interaction. (TIF) [file pone.0135293.s007.tif]

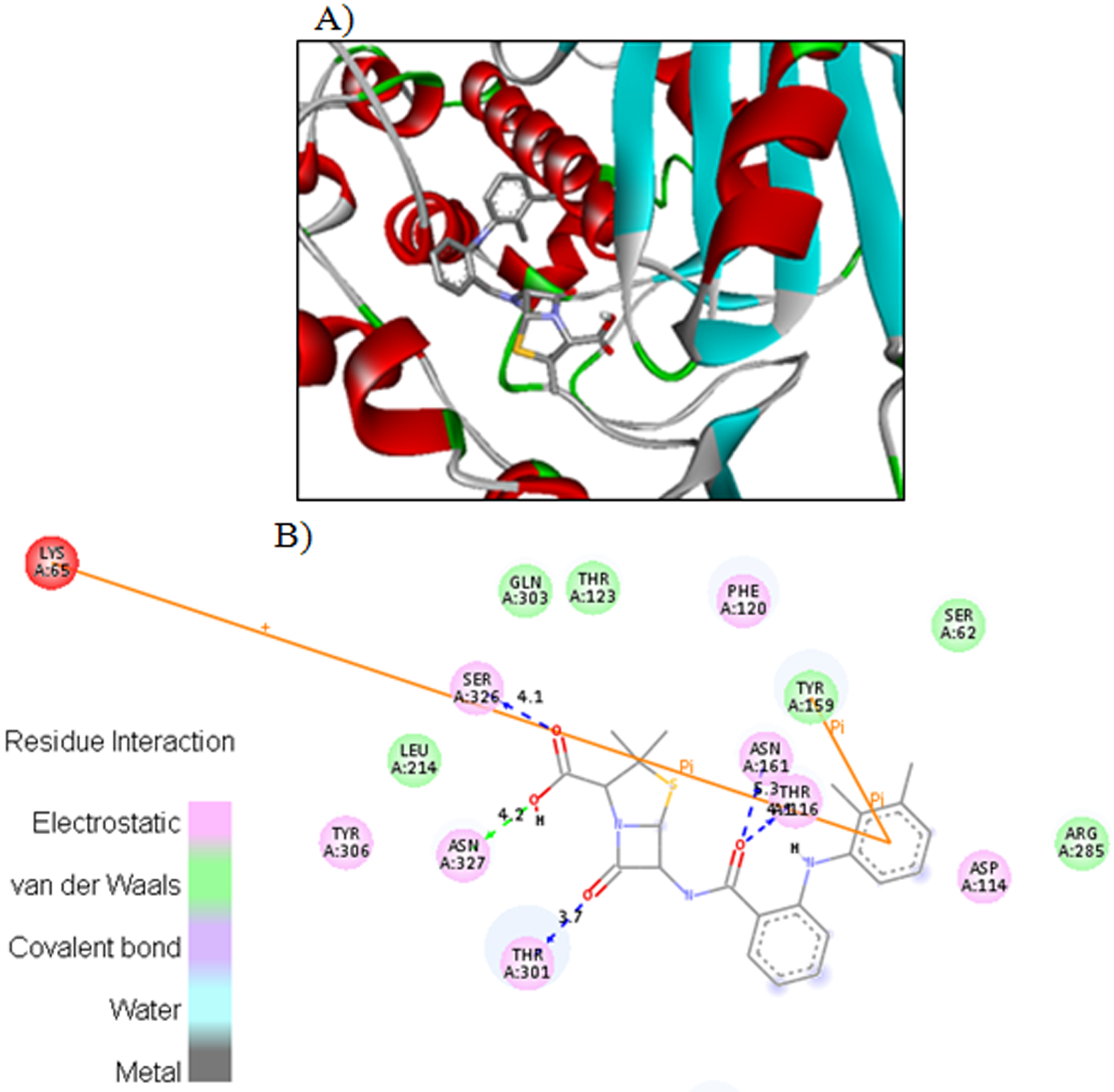

Supplement: S8 Fig — A) The three-dimensional docking of the compound 4h in the binding pocket. B) The two dimensional interactions of 4h with amino acid residues are shown as balls colored by the type of interaction. (TIF) [file pone.0135293.s008.tif]
