# Supplementary material for: Novel Penicillin Analogues as Potential Antimicrobial Agents; Design, Synthesis and Docking Studies
Source: PLoS One. 2015 Aug 12;10(8):e0135293. doi: 10.1371/journal.pone.0135293 (PMC4534092; doi:10.1371/journal.pone.0135293)
Supplement: S1 Table — (DOCX) [file pone.0135293.s009.docx]

**S1 Table** Descriptions of the various types of interactions between compounds and Penicilline Binding Protein (1CEF) functionalities

| **Element** | **Description** |
| --- | --- |
| 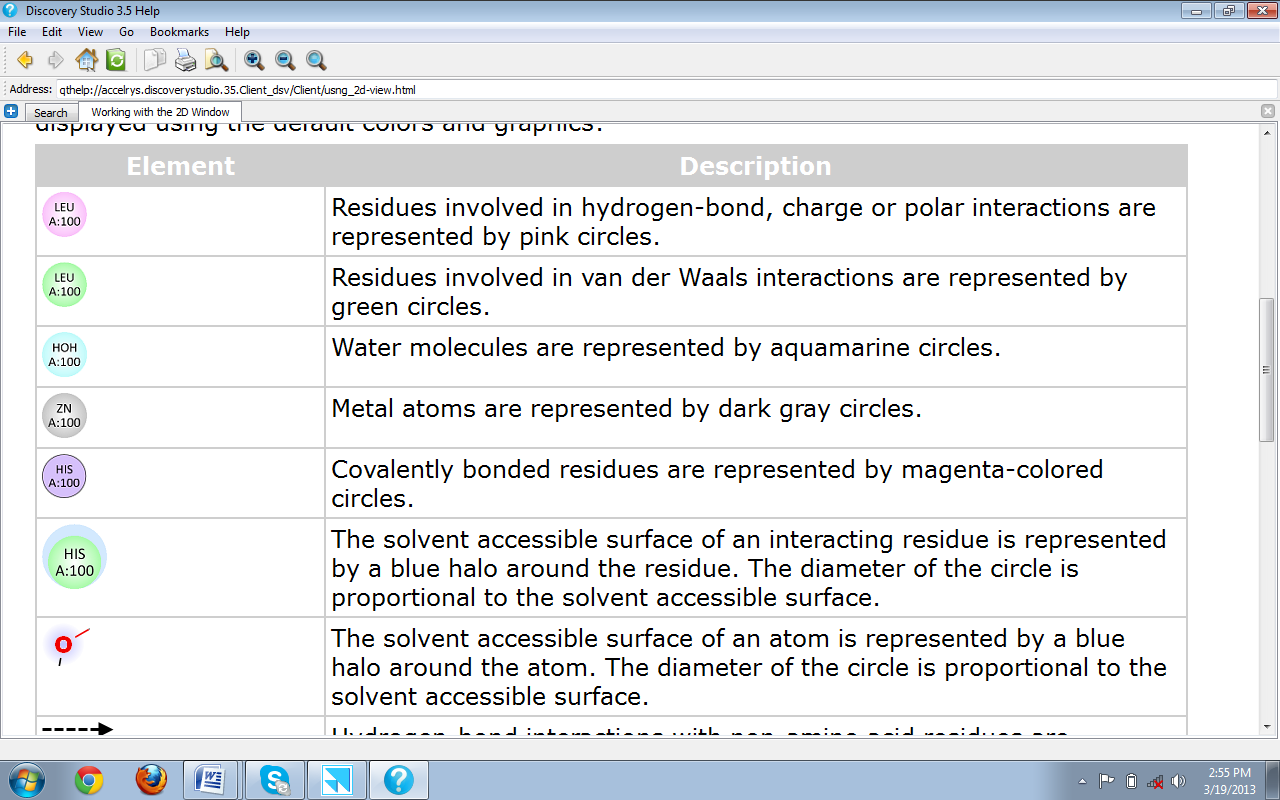 | Residues involved in hydrogen-bond, charge or polar interactions are represented by pink circles. |
| 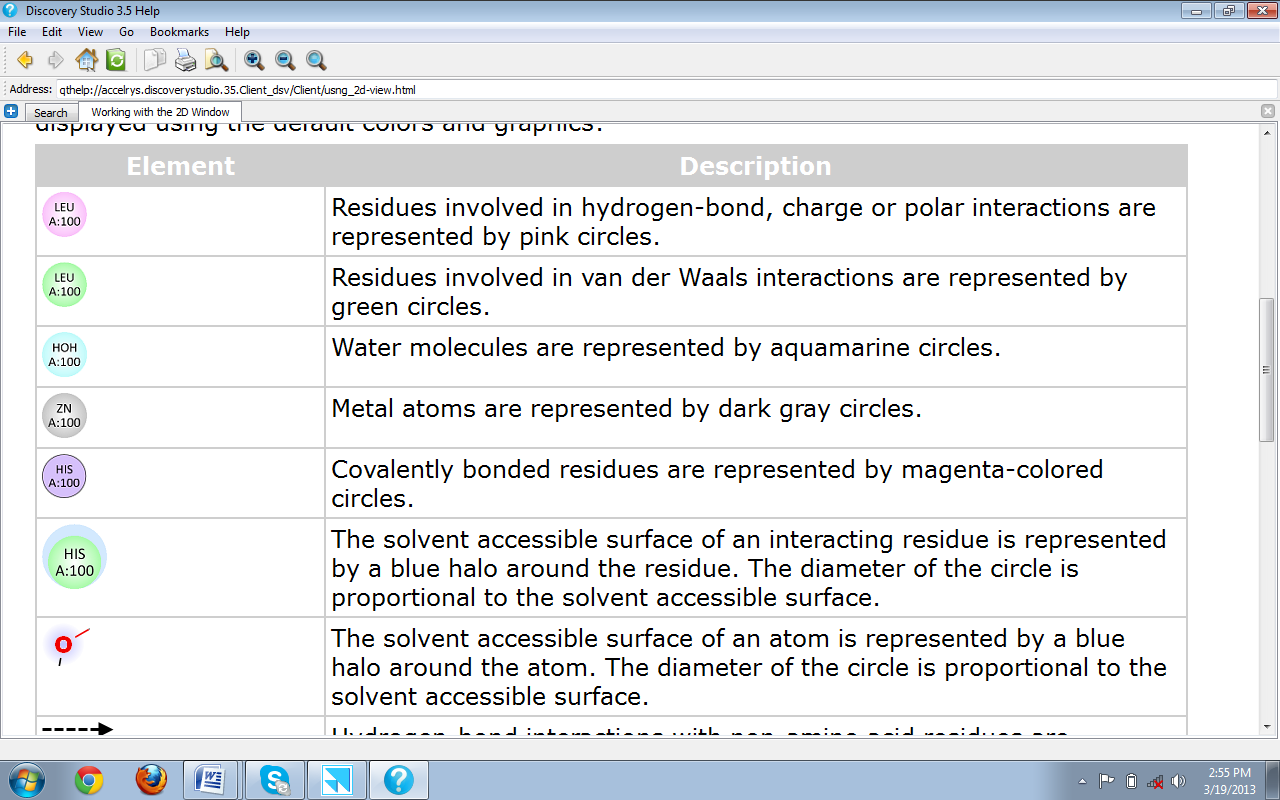 | Residues involved in van der Waals interactions are represented by green circles. |
| 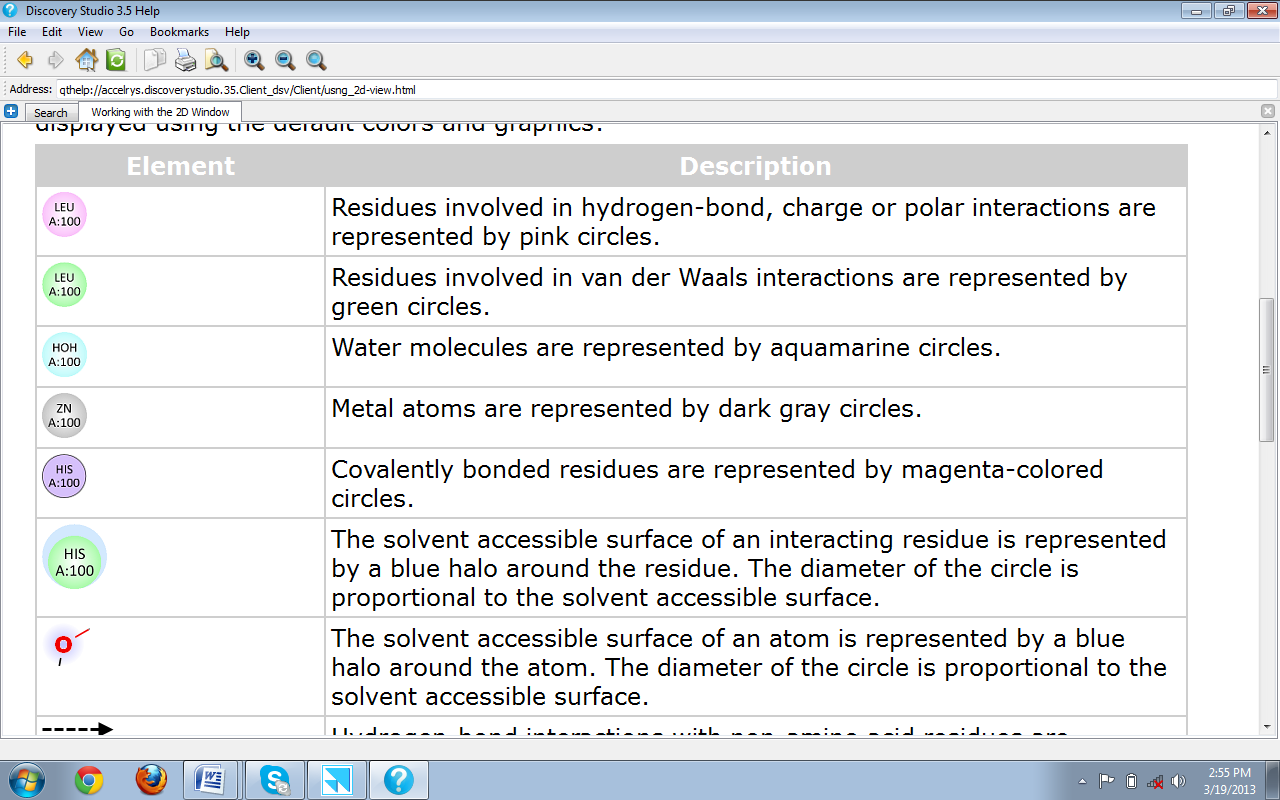 | Water molecules are represented by aquamarine circles. |
| 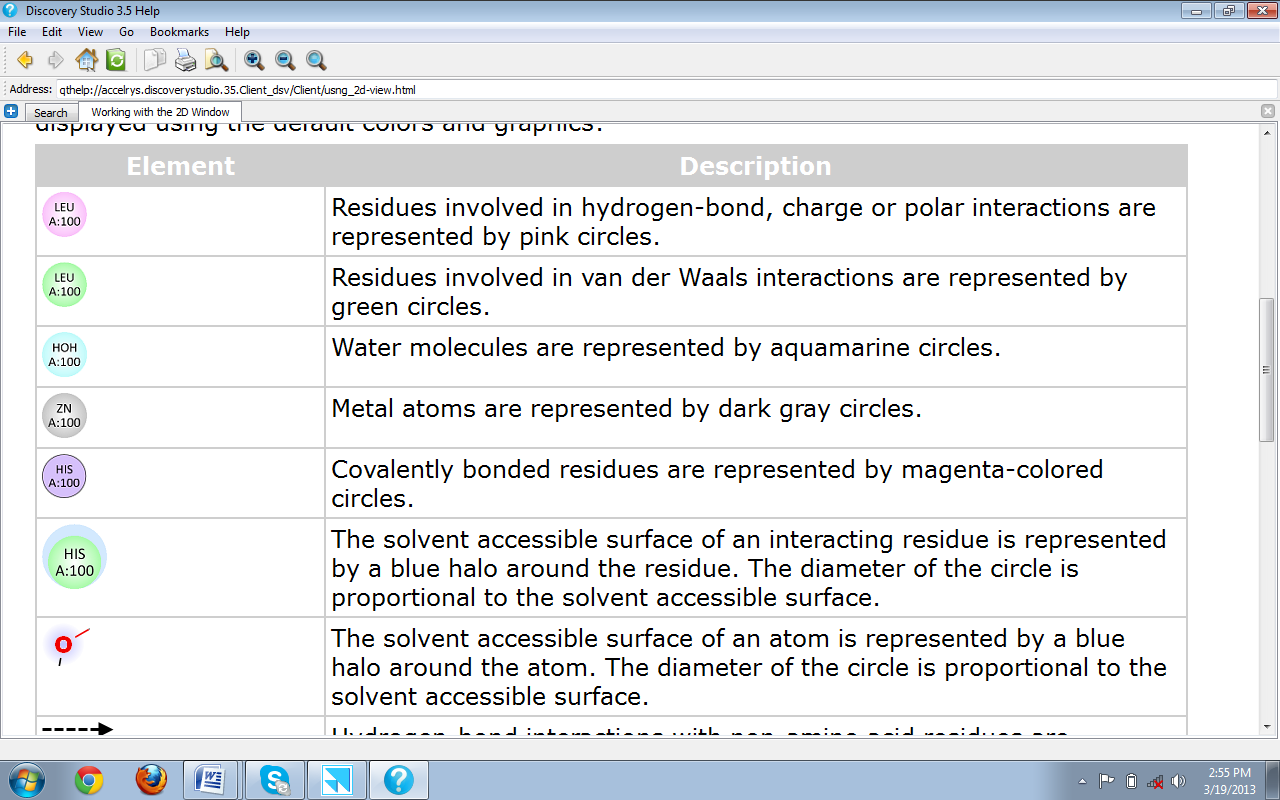 | Metal atoms are represented by dark gray circles. |
| 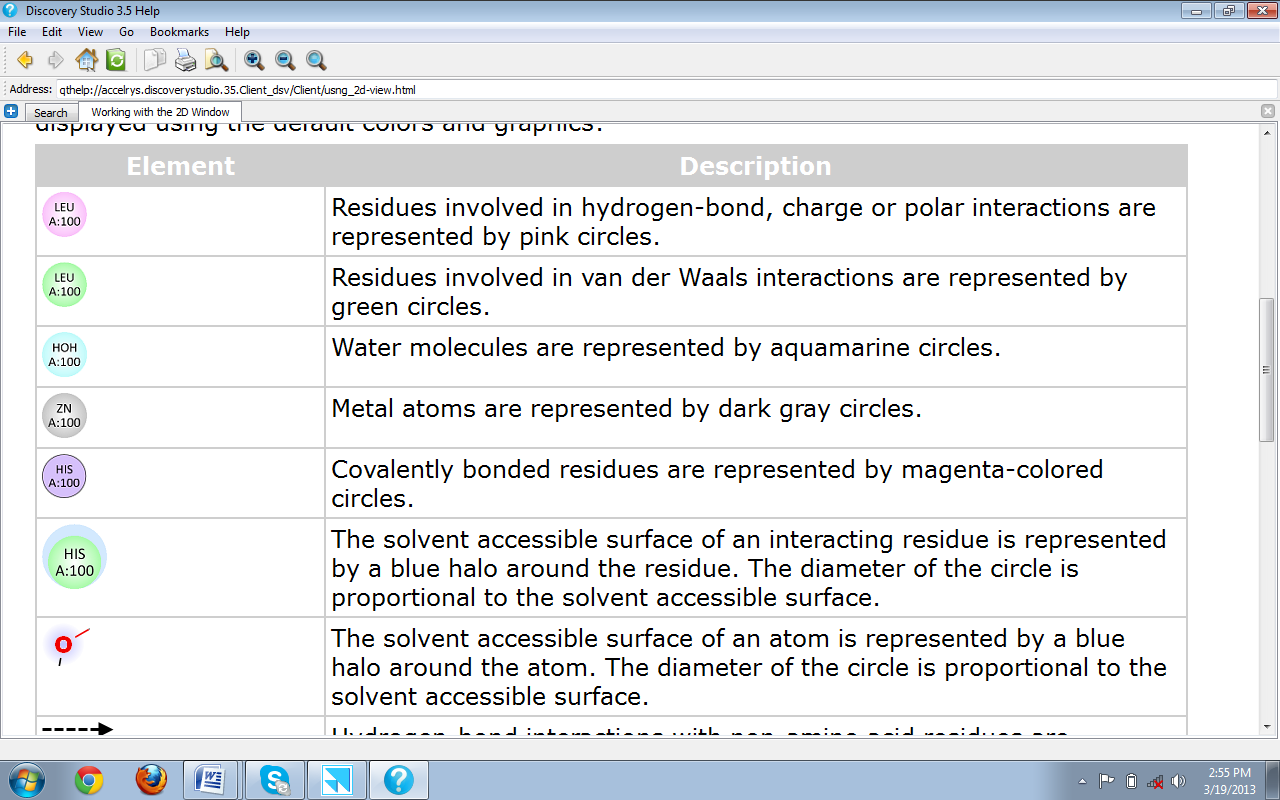 | Covalently bonded residues are represented by magenta-colored circles. |
| 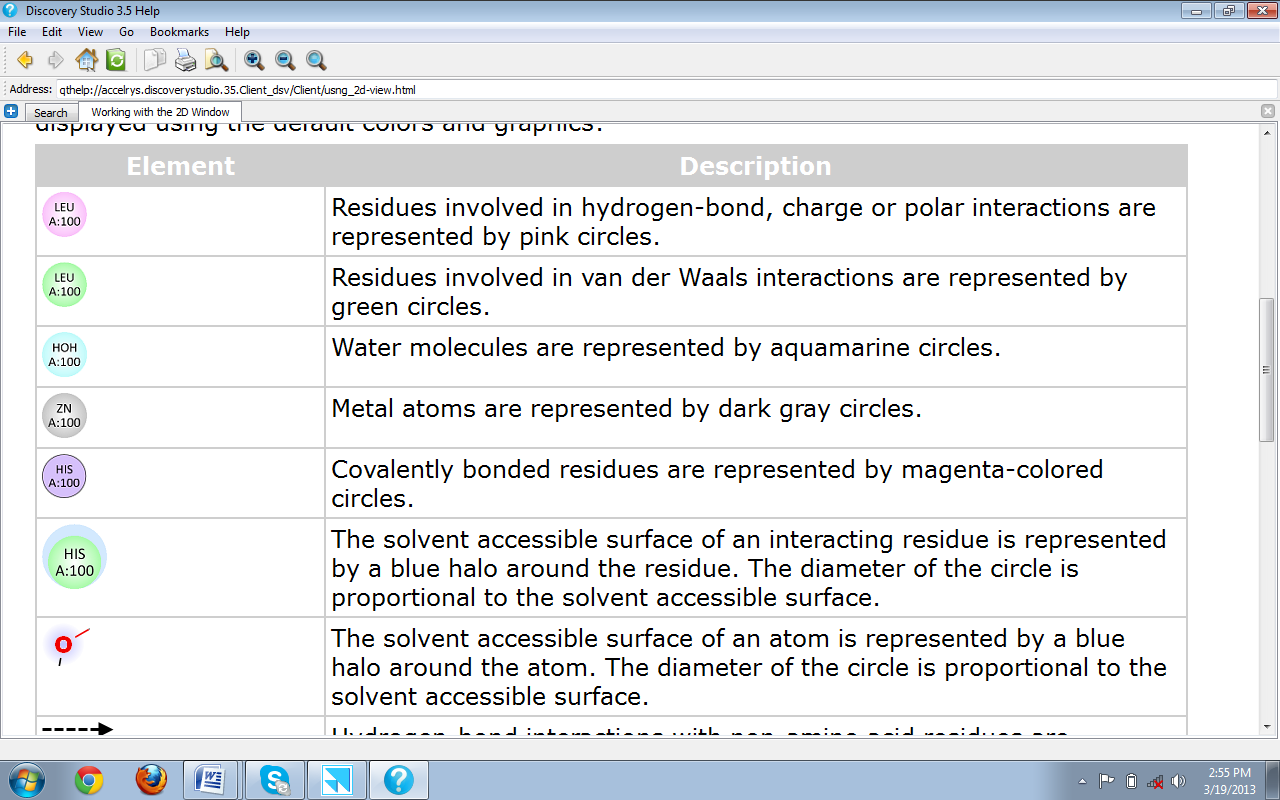 | The solvent accessible surface of an interacting residue is represented by a blue halo around the residue. The diameter of the circle is proportional to the solvent accessible surface. |
| 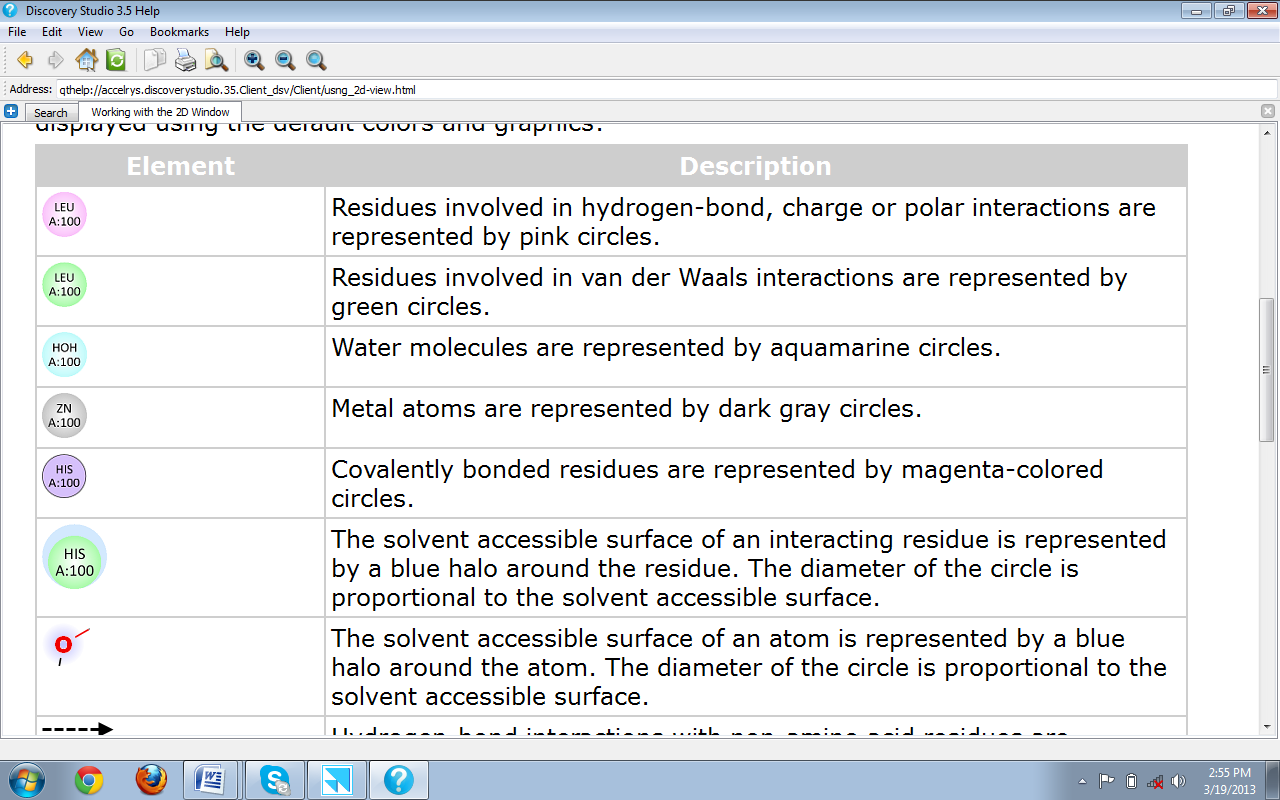 | The solvent accessible surface of an atom is represented by a blue halo around the atom. The diameter of the circle is proportional to the solvent accessible surface. |
| 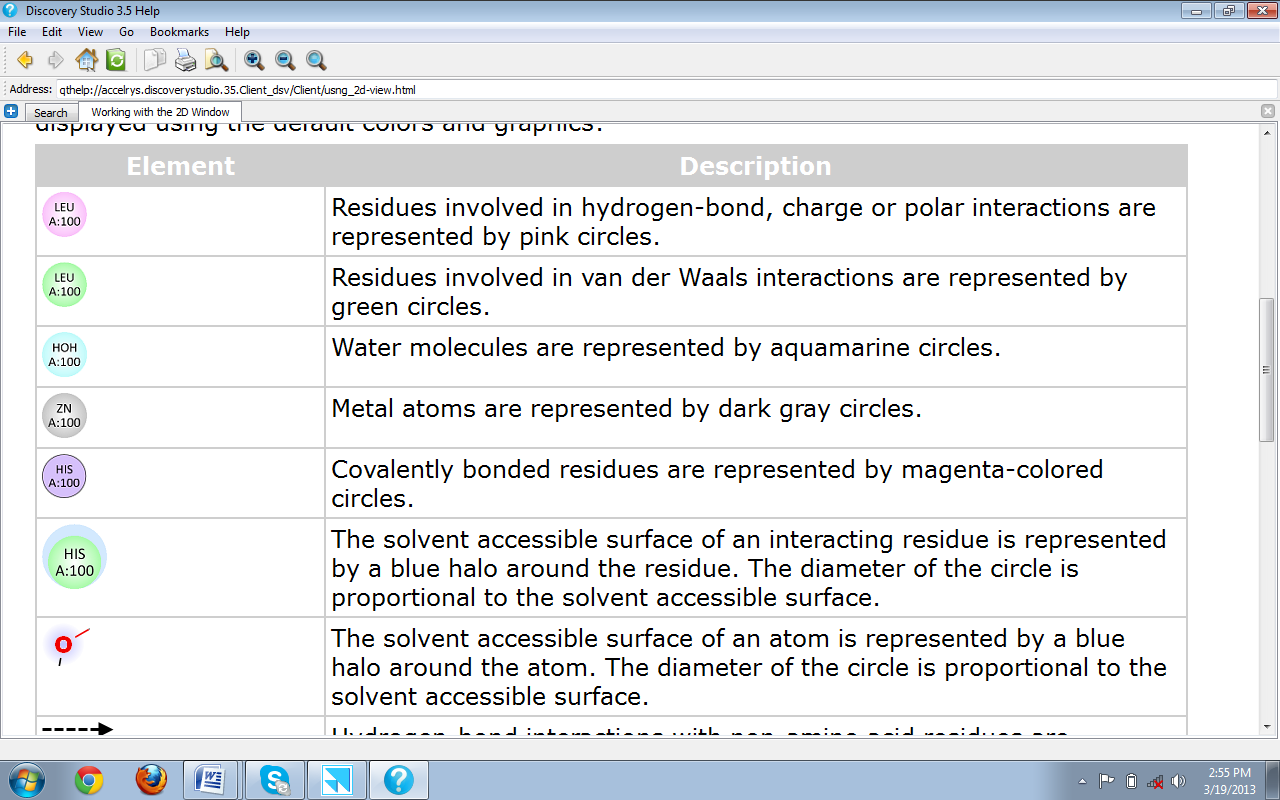 | Hydrogen-bond interactions with non-amino acid residues are represented by a black dashed line arrow directed towards the electron donor. |
| 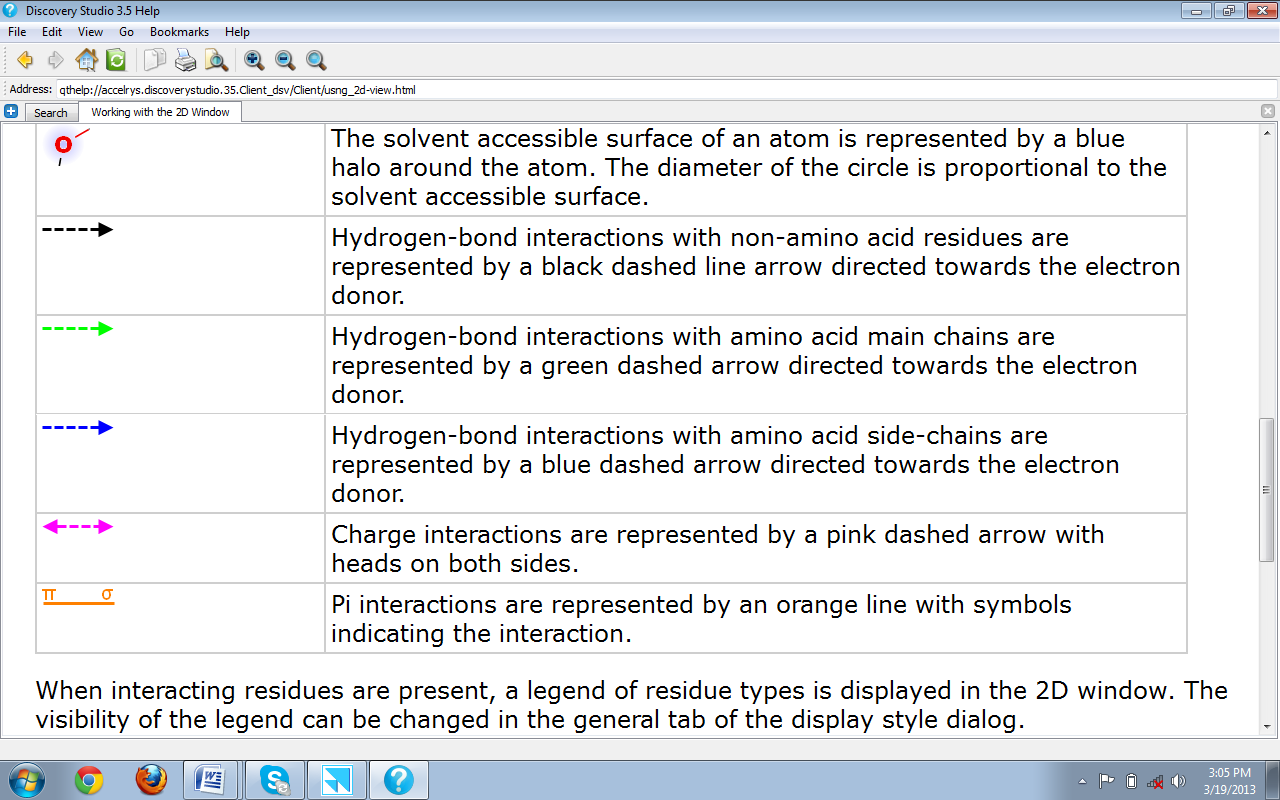 | Hydrogen-bond interactions with amino acid main chains are represented by a green dashed arrow directed towards the electron donor. |
| 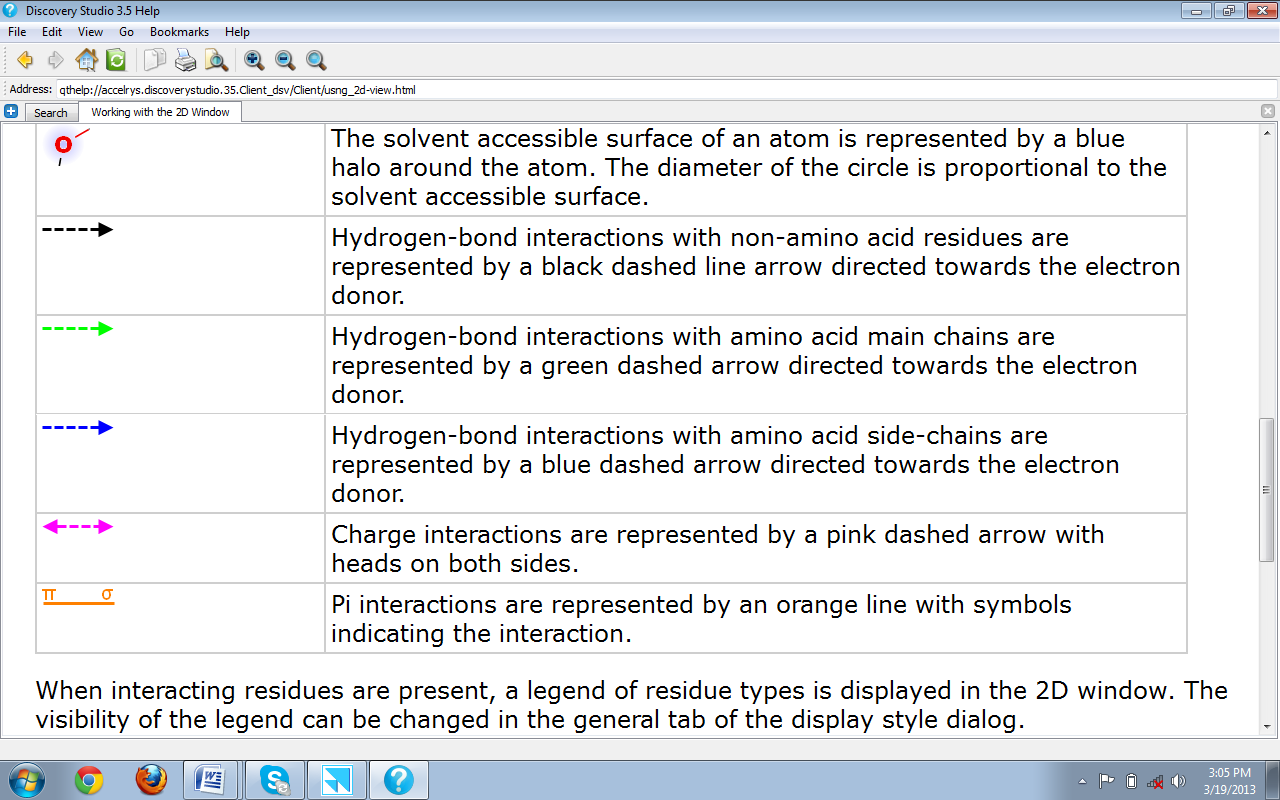 | Hydrogen-bond interactions with amino acid side-chains are represented by a blue dashed arrow directed towards the electron donor. |
| 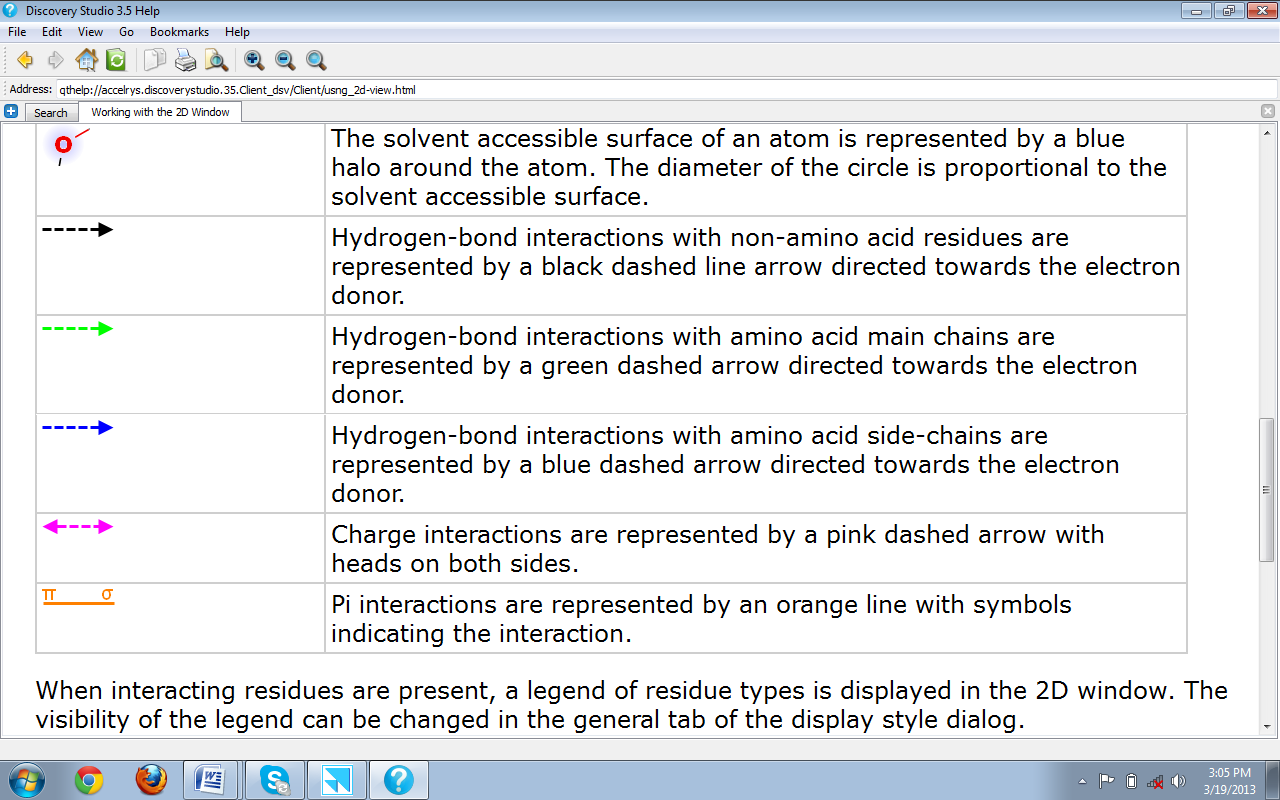 | Charge interactions are represented by a pink dashed arrow with heads on both sides. |
| 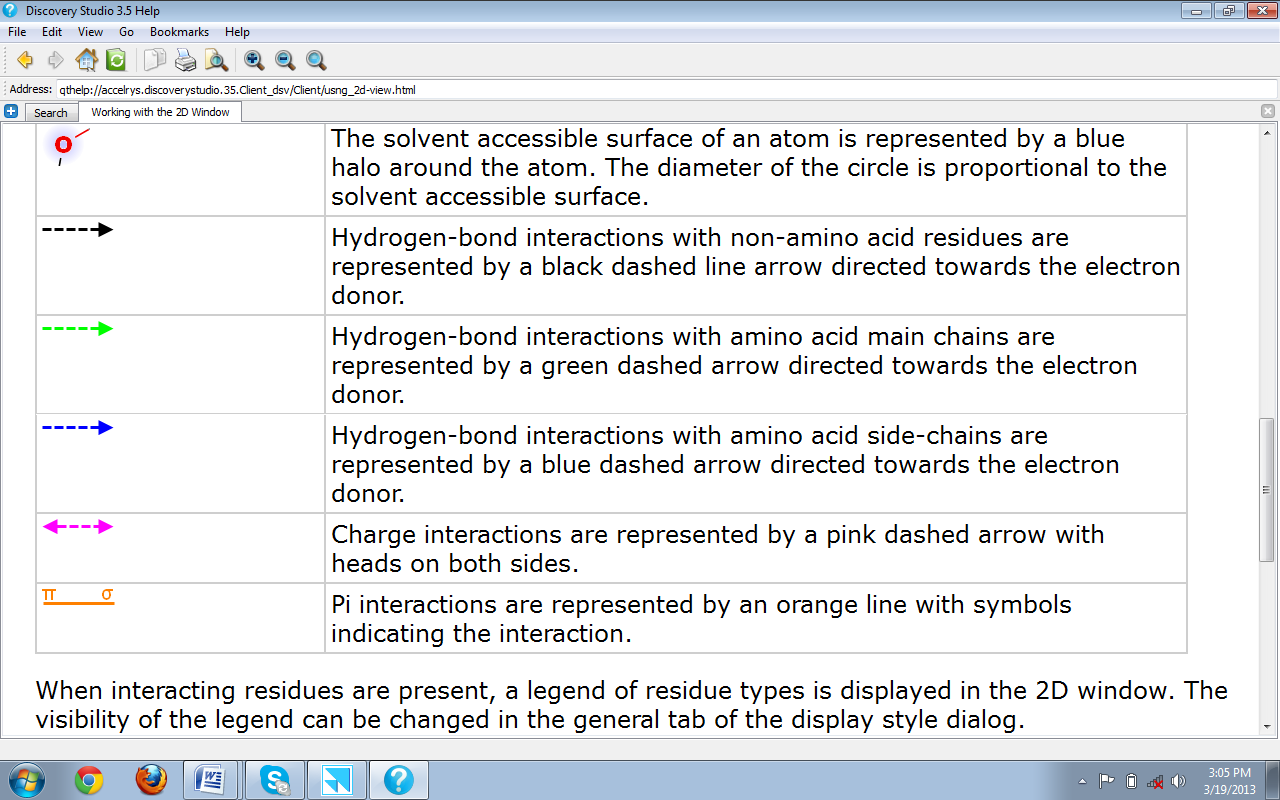 | Pi interactions are represented by an orange line with symbols indicating the interaction. |


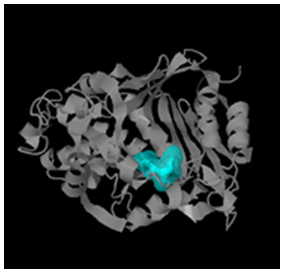


**S1 Fig.** Image showing the position of the active site occupied between five-stranded anti-parallel β-sheet and the large α-helical cluster.


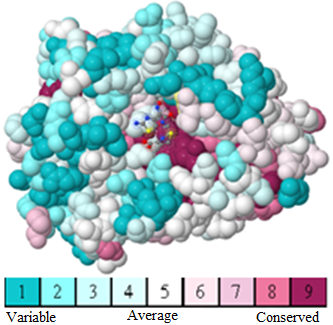


**S2 Fig.** The conserved amino acid residues of the receptor protein.


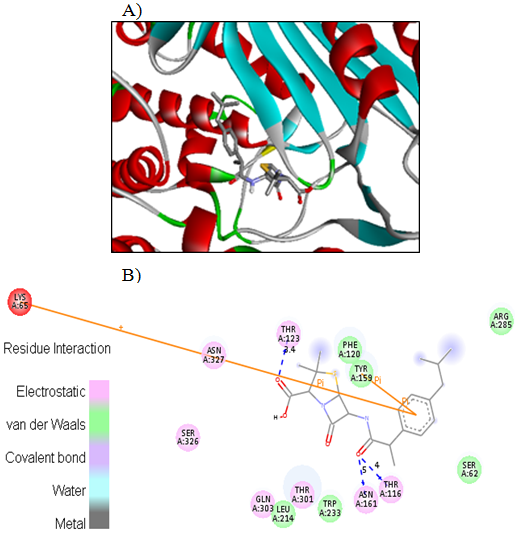


**S3 Fig.** The potential ligand-protein interactions of compound **4a** with the active site of Penicillin binding protein (PDB ID 1CEF) generated by using Discovery Studio 4.0. A) The three-dimensional docking of the compound **4a** in the binding pocket. B) The two dimensional interactions of **4a** with amino acid residues are shown as balls colored by the type of interaction.


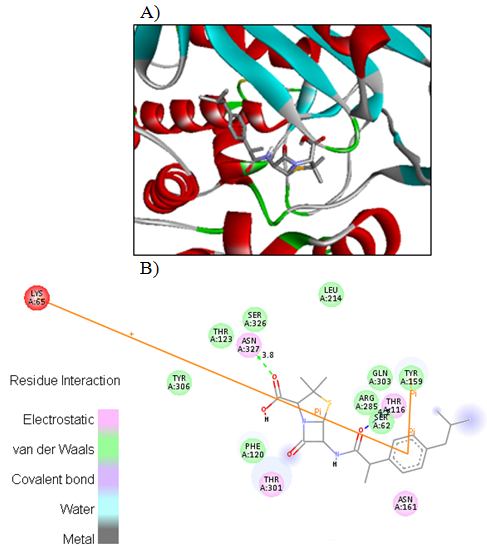


**S4 Fig.** The potential ligand-protein interactions of compound **4b** with the active site of Penicillin binding protein (PDB ID 1CEF) generated by using Discovery Studio 4.0. A) The three-dimensional docking of the compound **4b** in the binding pocket. B) The two dimensional interactions of **4b** with amino acid residues are shown as balls colored by the type of interaction.


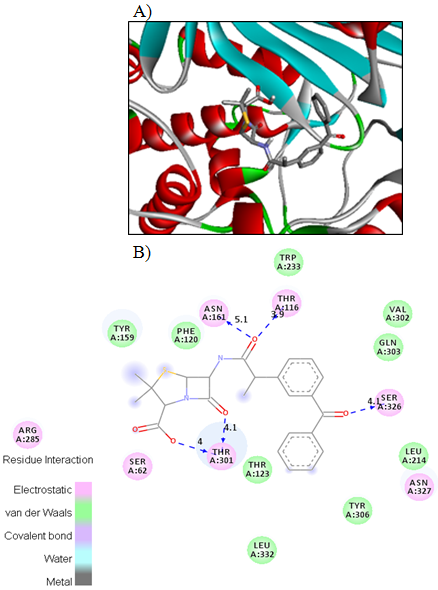


**S5 Fig.** The potential ligand-protein interactions of compound **4d** with the active site of Penicillin binding protein (PDB ID 1CEF) generated by using Discovery Studio 4.0. A) The three-dimensional docking of the compound **4d** in the binding pocket. B) The two dimensional interactions of **4d** with amino acid residues are shown as balls colored by the type of interaction.


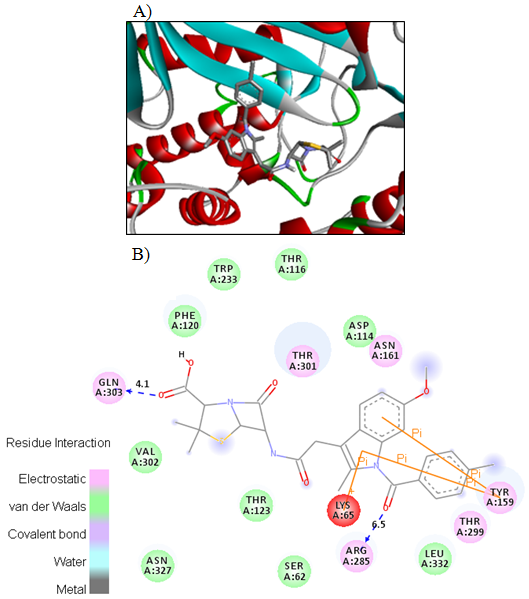


**S6 Fig.** The potential ligand-protein interactions of compound **4f** with the active site of Penicillin binding protein (PDB ID 1CEF) generated by using Discovery Studio 4.0. A) The three-dimensional docking of the compound **4f** in the binding pocket. B) The two dimensional interactions of **4f** with amino acid residues are shown as balls colored by the type of interaction.


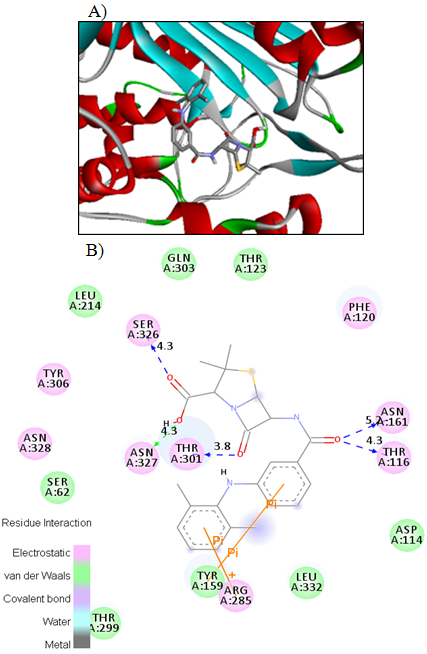


**S7 Fig.** The potential ligand-protein interactions of compound **4g** with the active site of Penicillin binding protein (PDB ID 1CEF) generated by using Discovery Studio 4.0. A) The three-dimensional docking of the compound **4g** in the binding pocket. B) The two dimensional interactions of **4g** with amino acid residues are shown as balls colored by the type of interaction.


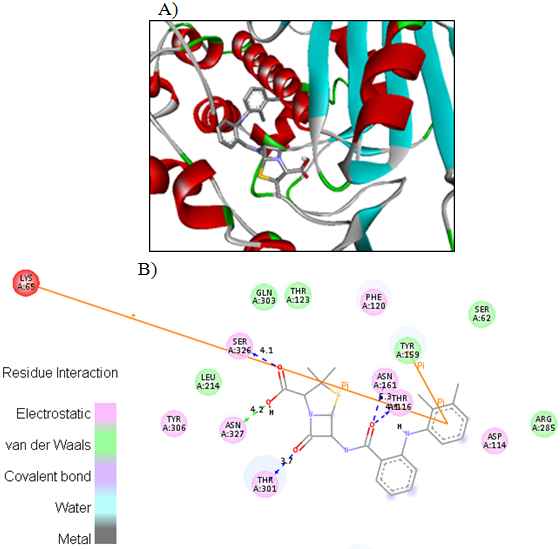


**S8 Fig.** The potential ligand-protein interactions of compound **4h** with the active site of Penicillin binding protein (PDB ID 1CEF) generated by using Discovery Studio 4.0. A) The three-dimensional docking of the compound **4h** in the binding pocket. B) The two dimensional interactions of **4h** with amino acid residues are shown as balls colored by the type of interaction.
